# Supplementary material for: Associations of active travel with adiposity among children and socioeconomic differentials: a longitudinal study
Source: BMJ Open. 2021 Jan 12;11(1):e036041. doi: 10.1136/bmjopen-2019-036041 (PMC7805367; doi:10.1136/bmjopen-2019-036041)
Supplement: Supplementary data [file bmjopen-2019-036041supp001.pdf]

Appendix table 1: Mode of travel to school in the Millennium Cohort Study in 2011/12, age 10-11 years

		Car	PT	Active
Gender	Male	47.4	5.3	47.3
	Female	47.2	5.2	47.6
Country	England	45.5	2.6	51.9
	Wales	51.1	7.6	41.3
	Scotland	37.8	9.8	52.4
	Northern Ireland	62.9	13.9	23.1
NS-SEC social class	Not economically active	28.7	4.9	66.4
	Lower	39.7	5.7	54.6
	Intermediate	53.7	4.9	41.4
	Managerial/professional	53.7	5.1	41.2
Household income group	Lowest	34.6	5.5	59.9
	Second lowest	43.3	6.6	50.0
	Middle	47.8	5.2	47.0
	Second highest	51.1	4.5	44.4
	Highest	56.2	4.7	39.1
Ethnic group	White	48.0	5.7	46.3
	Mixed	41.6	4.7	53.7
	Indian	58.4	1.0	40.6
	Pakistani or Bangladeshi	38.1	1.1	60.8
	Black or Black British	40.5	6.2	53.3
	Other	43.5	3.3	53.3
Growth spurt*	Not yet/barely started	-	-	-
	Begun or completed	-	-	-
Breakfast consumption	< 7 days per week	42.9	7.3	49.8
	Every day	47.6	5.1	47.3
Portions of fruit per day	≤3 portions per day	46.1	5.1	48.8
	≥2 portions per day	47.6	5.3	47.1
Physical activity	≤2 days per week	48.9	4.2	46.9
	3-4 days per week	50.1	5.0	44.9
	≥ 5 days per week	46.2	5.7	48.1
TV or computer use	≤2 hours per day	47.5	5.5	47.0
	≥3 hours per day	46.4	4.2	49.4
Overall		47.3	5.3	47.4

PT = Public Transport , * N/A

Appendix table 2: Mode of travel to school in the Millennium Cohort Study in 2007/08, age 7-8 years

		Car	PT	Active
Gender	Male	42.6	6.6	50.9
	Female	44.7	6.9	48.4
Country	England	42.1	4.7	53.2
	Wales	47.1	8.4	44.5
	Scotland	32.2	10.1	57.8
	Northern Ireland	60.8	13.6	25.6
NS-SEC social class	Not economically active	36.8	6.8	56.4
	Lower	38.3	5.9	55.7
	Intermediate	47.8	6.4	45.8
	Managerial/professional	50.7	7.4	41.8
Household income group	Lowest	31.0	6.4	62.6
	Second lowest	40.2	8.2	51.7
	Middle	48.5	6.8	44.7
	Second highest	48.7	6.0	45.3
	Highest	46.3	6.4	47.3
Ethnic group	White	43.9	6.9	49.2
	Mixed	42.2	10.1	47.7
	Indian	55.3	2.6	42.1
	Pakistani or Bangladeshi	41.3	1.8	56.9
	Black or Black British	33.6	12.3	54.0
	Other	40.0	5.6	54.4
Growth spurt	Not yet/barely started	45.0	6.5	48.5
	Begun or completed	42.3	7.0	50.7
Breakfast consumption	< 7 days per week	40.0	8.5	51.5
	Every day	44.2	6.5	49.3
Portions of fruit per day	≤3 portions per day	42.3	7.3	50.5
	≥2 portions per day	44.3	6.5	49.2
Physical activity	≤2 days per week	44.7	6.8	48.5
	3-4 days per week	44.4	6.6	49.0
	≥ 5 days per week	42.7	6.7	50.6
TV or computer use	≤2 hours per day	44.5	7.1	48.4
	≥3 hours per day	39.9	5.0	55.1
Overall		43.7	6.7	49.6

Appendix Table 3: Results from longitudinal fixed-effects regression of impacts of switching school travel mode and BMI stratified by markers of Socio-Economic Position

NSSEC groups	Coefficient	Lower CI	Upper CI
Managerial/professional	-0.09	-0.22	0.04
Self-employed intermediate	-0.22	-0.37	-0.07
Lower semi-routine routine	-0.21	-0.37	-0.05
Not economically active	-0.33	-0.55	-0.11
Household income groups			
Highest quantile income	-0.11	-0.24	0.03
Fourth quantile	-0.09	-0.23	0.04
Third quantile	-0.17	-0.34	0.00
Second quantile	-0.35	-0.53	-0.16
Lower quantile income	-0.32	-0.58	-0.06

NSSEC = National Statistics Socio-economic Classification, CI = Confidence Interval

Models adjusted for individual-level fixed effects and changes in: country; highest NSSEC in household; household income; eating breakfast; frequency of eating breakfast; self-reported growth spurt; hours of TV and computer use per day; and days per week of moderate-to-vigorous physical activity.

Appendix Table 4: Results from longitudinal fixed-effects regression of impacts of switching school travel mode and percentage body fat stratified by markers of Socio-Economic Position

NSSEC groups	Coefficient	Lower CI	Upper CI
Managerial/professional	-0.43	-0.78	-0.08
Self-employed intermediate	-0.59	-0.99	-0.20
Lower semi-routine routine	-0.47	-0.85	-0.08
Not economically active	-0.78	-1.27	-0.29
Household income groups			
Highest quantile income	-0.55	-1.01	-0.09
Fourth quantile	-0.41	-0.86	0.04
Third quantile	-0.60	-1.12	-0.08
Second quantile	-1.29	-1.93	-0.64
Lower quantile income	-0.71	-1.47	0.05

Models adjusted for individual-level fixed effects and changes in: country; highest NSSEC in household; household income; eating breakfast; frequency of eating breakfast; self-reported growth spurt; hours of TV and computer use per day; and days per week of moderate-to-vigorous physical activity.

Appendix table 5: Fully adjusted odds ratios travelling 15 minutes or more each way by walking or cycling

		%	OR	Lower CI	Upper CI
Gender	Male	42.2	ref	ref	ref
	Female	45.1	1.14	1.00	1.30
Country	England	45.2	ref	ref	ref
	Wales	38.5	0.65	0.53	0.79
	Scotland	41.2	0.74	0.61	0.91
	Northern Ireland	36.6	0.60	0.43	0.83
NS-SEC social class	Not economically active	39.7	ref	ref	ref
	Lower	43.8	1.10	0.91	1.33
	Intermediate	44.5	1.07	0.87	1.32
	Managerial/professional	47.0	1.20	0.98	1.47
Household income group	Lowest	38.2	ref	ref	ref
	Second lowest	43.8	1.00	0.80	1.25
	Middle	44.9	0.98	0.77	1.23
	Second highest	44.9	0.88	0.69	1.11
	Highest	46.0	0.86	0.66	1.11
Ethnic group	White	46.2	ref	ref	ref
	Mixed	53.8	1.30	0.67	2.53
	Indian	36.4	0.60	0.41	0.88
	Pakistani or Bangladeshi	23.9	0.30	0.23	0.40
	Black or Black British	38.9	0.66	0.45	0.97
	Other	32.3	0.53	0.31	0.90
Growth spurt	Not yet/barely started	43.3	ref	ref	ref
	Begun or completed	44.2	0.99	0.87	1.13
Breakfast consumption	< 7 days per week	43.3	ref	ref	ref
	Every day	44.0	1.07	0.94	1.22
Portions of fruit per day	≤3 portions per day	43.4	ref	ref	ref
	≥2 portions per day	44.4	1.00	0.87	1.16
Physical activity	≤2 days per week	42.7	ref	ref	ref
	3-4 days per week	45.6	1.14	0.97	1.34
	≥ 5 days per week	42.5	0.98	0.83	1.15
TV or computer use	≤2 hours per day	42.5	ref	ref	ref
	≥3 hours per day	43.9	1.01	0.86	1.18
