# Supplementary material for: Associations of active travel with adiposity among children and socioeconomic differentials: a longitudinal study
Source: BMJ Open. 2021 Jan 12;11(1):e036041. doi: 10.1136/bmjopen-2019-036041 (PMC7805367; doi:10.1136/bmjopen-2019-036041)
Supplement: Author's manuscript [file bmjopen-2019-036041.draft_revisions.pdf]

BMJ Open is committed to open peer review. As part of this commitment we make the peer review history of every article we publish publicly available.

When an article is published we post the peer reviewers' comments and the authors' responses online. We also post the versions of the paper that were used during peer review. These are the versions that the peer review comments apply to.

The versions of the paper that follow are the versions that were submitted during the peer review process. They are not the versions of record or the final published versions. They should not be cited or distributed as the published version of this manuscript.

BMJ Open is an open access journal and the full, final, typeset and author-corrected version of record of the manuscript is available on our site with no access controls, subscription charges or pay-per-view fees (http://bmjopen.bmj.com).

If you have any questions on BMJ Open's open peer review process please email
info.bmjopen@bmj.com

BMJ Open

Associations of active travel with adiposity among children and socio-economic differentials: a longitudinal study

Journal:	*BMJ Open*
Manuscript ID	bmjopen-2019-036041
Article Type:	Original research
Date Submitted by the Author:	27-Nov-2019
Complete List of Authors:	Laverty, Anthony; Imperial College London, Primary Care and Public Health<br>Hone, Thomas; Imperial College London,<br>Goodman, Anna; London School of Hygiene and Tropical Medicine, Faculty of Epidemiology and Population Health<br>Kelly, Yvonne; UCL, Epidemiology and Public Health<br>Millett, Christopher; Imperial College, Primary Care and Social Medicine
Keywords:	PUBLIC HEALTH, Community child health < PAEDIATRICS, PREVENTIVE MEDICINE

SCHOLARONE™
Manuscripts

I, the Submitting Author has the right to grant and does grant on behalf of all authors of the Work (as defined in the below author licence), an exclusive licence and/or a non-exclusive licence for contributions from authors who are: i) UK Crown employees; ii) where BMJ has agreed a CC-BY licence shall apply, and/or iii) in accordance with the terms applicable for US Federal Government officers or employees acting as part of their official duties; on a worldwide, perpetual, irrevocable, royalty-free basis to BMJ Publishing Group Ltd ("BMJ") its licensees and where the relevant Journal is co-owned by BMJ to the co-owners of the Journal, to publish the Work in this journal and any other BMJ products and to exploit all rights, as set out in our licence.

The Submitting Author accepts and understands that any supply made under these terms is made by BMJ to the Submitting Author unless you are acting as an employee on behalf of your employer or a postgraduate student of an affiliated institution which is paying any applicable article publishing charge ("APC") for Open Access articles. Where the Submitting Author wishes to make the Work available on an Open Access basis (and intends to pay the relevant APC), the terms of reuse of such Open Access shall be governed by a Creative Commons licence – details of these licences and which Creative Commons licence will apply to this Work are set out in our licence referred to above.

Other than as permitted in any relevant BMJ Author's Self Archiving Policies, I confirm this Work has not been accepted for publication elsewhere, is not being considered for publication elsewhere and does not duplicate material already published. I confirm all authors consent to publication of this Work and authorise the granting of this licence.

For peer review only

Associations of active travel with adiposity among children and socio-economic differentials: a longitudinal study

Anthony A. Laverty[1*], Thomas Hone[1], Anna Goodman[2], Yvonne Kelly[3], Christopher Millett[1]

[1] Public Health Policy Evaluation Unit, School of Public Health, Imperial College London

[2] Faculty of Epidemiology and Population Health, London School of Hygiene and Tropical Medicine

[3] Department of Epidemiology and Public Health, 1-19 Torrington Place, University College London, London, WC1E 6BT, UK

* Corresponding author a.laverty@imperial.ac.uk

For peer review only

Objectives

We examined longitudinal associations between modes of travel to school and adiposity among children.

Setting

UK

Participants

8,432 children surveyed at ages 7, 11 and 14 years from the UK Millennium Cohort study.

Primary and secondary outcomes

Objective percentage body fat and Body Mass Index (BMI). Transport mode was categorised as private motorised transport, public transport, active transport (walking or cycling). SEP was measured by household income group and occupational social class (National Statistics Socio-Economic Classification). Fixed effects longitudinal regression examined associations in changes in mode of travel to school and adiposity, controlling for both time-varying and time-invariant potential confounders. Interaction tests and stratified analyses investigated differences by markers of SEP.

Results

At age 14, 25.8% of children reported using private motorised transport, 34.8% used public transport, and 39.4% used active transport to get to school. Compared with continuing to use private transport, switching to active transport was associated with a lower BMI (-0.21kg/m^2, 95% Confidence Interval -0.31;-0.10) and body fat (-0.55%, -0.80;-0.31). Switching to public transport was

For peer review only - http://bmjopen.bmj.com/site/about/guidelines.xhtml

associated with lower percentage body fat (-0.43%, -0.75;-0.12), but associations with BMI did not reach statistical significance (-0.13kg/m2, -0.26; 0.01). Interaction tests showed a trend for these effects to be stronger in more deprived groups, but these interactions did not reach statistical significance

Conclusion

This longitudinal study from a key lifecourse period concluded that switching to physically active forms of travel can have beneficial adiposity impacts and these associations may be more apparent for more disadvantaged children. Increasing active travel has potential to ameliorate inequalities.

For peer review only

Strengths and limitations of this study

- Used an established and representative UK birth cohort study with high quality data following up the same children over 8 years.
- We used two objective measures of adiposity and employed models that produce robust estimates of within-individual changes and were able to adjust for unobserved time-invariant individual characteristics
- However, we rely on self-reported mode of travel to school, which did not capture information on multi-mode journeys or variation in different modes across the week.
- There was an average of three years between data collection points, and we do not know at what point between data collections individuals changed their travel behaviour.
- While we were able to consider a wide range of covariates, there may be some residual confounding from time-varying factors not considered such as choosing to lead a healthier lifestyle in other ways also, and changes in adiposity may be occurring via alternative mechanisms than active travel.

For peer review only

Introduction

Insufficient physical activity is associated with increased cardio-metabolic risk, including higher levels of adiposity in children as well as adults [1]. Children are an important group for physical activity promotion, both because objective data has found that the majority of children do not reach recommended levels, and because there is evidence that levels of physical activity track across the lifecourse, meaning that those who are insufficiently active early in life are at risk of this continuing into later life [2][3][4][5]. The period of transition from primary to secondary education is characterised by significant changes to children's social and physical environment which can strongly influence health behaviours [6].

Active travel (that is, the use of non-motorised modes of travel) is one way to increase physical activity and reduce adiposity [7]. The use of active travel in children has been linked to higher levels of physical activity in a range of settings, including during the journey to school [5][8][9][10]. Modes of travel change significantly during childhood, with the majority of travel being with caregivers in primary school and then changing to more independent modes later [11]. There are also significant differentials across socio-economic position (SEP) in physical activity profiles, use of active travel, and cardio-metabolic health risks in children, which together contribute to levels of health inequalities between groups [12][13][14]. While active travel may have the potential to offset some of these inequalities, a recent review by Sport England concluded that there is a comparative lack of evidence on the impacts of active travel across groups across markers of SEP [15].

Much of the evidence on the impacts of active travel has been cross-sectional and there has been a lack of evidence using large-scale prospective studies, especially in children. Studies have highlighted conflicting evidence on the potential role of active travel to school on body composition and adiposity and, a recent systematic review found a limited number of prospective studies in children and did not identify an association between active travel and obesity in children [16][17][18][19]. This study adds to the evidence base by using a robust analytical strategy on data from a representative cohort of children during a key period in their development to investigate the role of active travel to school on objectively measured adiposity, and the potential for differential effects according to two different measures of SEP.

Methods

Sample and data

For peer review only

Data for this study came from the Millennium Cohort Study (MCS), which is a birth cohort study in the UK of children born between September 2000 and January 2002, sampled at approximately 9 months old [20]. Children were initially sampled in the year 2000 and follow up data is currently available from six sweeps. Sampling was based on a cluster-stratified framework with smaller population groups being over sampled, including those in the smaller nations of the UK, as well as those in disadvantaged areas and from ethnic minority backgrounds.

These analyses use data from the fourth sweep of data collection when children were aged 7/8 years old (response rate 82%), the fifth sweep of data collection children were 10/11 years old (response rate 81%), and the sixth sweep of data when aged 14/15 years old (response rate 76%). A range of topics covering the economic, social and health circumstances of children are covered alongside objective adiposity measurements. We focused on these sweeps of data as levels of active travel were low before the age of 7 years. Questionnaires and objective measures are administered by trained interviewers to standardised protocols and further details are available from http://www.cls.ioe.ac.uk/ [20]. Specific ethical approval was not required for this study as it involved only the secondary analyses of data that does not contain identifiable information.

Variables

This study used two objective measures of adiposity at all three time points: Body Mass Index (BMI) and percentage body fat. Height was measured using a Leicester stadiometer, and weight and body fat were measured using Tanita BF-522 W scales [20].

The exposure for this study was mode of travel to school assessed by asking caregivers "*How does (child) usually travel to school?",* with prompts to give the mode with longest distance if more than one mode was used. There were seven potential responses: Public transport; School or local authority bus; minibus or coach; Car or other vehicle (including Taxi); Bicycle; Walking; and Other. We categorised these responses into three categories of private motorised (Car or other vehicle (including Taxi)), public transport (Public transport; School or local authority bus; minibus or coach) and active travel (walking and cycling). We excluded "Other" responses (253 at age 14 (2.2%), 1,203 at age 11 (9.0%), 174 (1.3% at age 7)).

A range of covariates were included as potential confounders of the relationship between travel mode and adiposity, covering socioeconomic factors and health behaviours. Included covariates from all ages were sex; country of residence (England, Wales, Northern Ireland, Scotland); self-reported ethnicity; portions of fruit consumed per day (<3 vs. ≥3 pieces per day); breakfast consumption (seven days per week vs. not); screen use (hours per weekday watching TV or using a

For peer review only - http://bmjopen.bmj.com/site/about/guidelines.xhtml

computer categorised as <3hours vs. ≥ 3 hours). We also created a variable to reflect days per week of sport/exercise each week (categorised as < once a week, 1–2 times a week, 3+ times a week). We also used data on whether caregivers report that their child has experienced a growth spurt, assessed using the question "*Most children have a growth spurt as they approach and during their teens. Would you say that [Cohort child's name]'s growth spurt ....?"* This question was asked only at ages 11 and 14 and we categorised this into two categories of has definitely started vs. not. We used two markers of SEP: household income group (equivalised based on the Organisation for Economic Co-operation and Development and categorised in five groups) and household occupational social class measured using the National Statistics Socio-Economic Classification (NS-SEC; categorised as *Not economically active; Lower; Intermediate; Managerial/Professional*).

For longitudinal analyses we included children who had complete data available from ages 7 to 14. For cross-sectional analyses at age 14 there were 11,714 children in the sample, of which we used data from 10,722 (91.5%) after excluding 253 (2.2%) for using an "other" form of transport and 739 (6.3%) for missing data on relevant covariates. For longitudinal analyses we include data from 8,432 children (82.5% of those with complete data at age 14) who had data on all relevant data from ages 7 to 14.

Analyses

The distribution of modes of travel to school across socio-demographic and behavioural characteristics was first examined descriptively.

Longitudinal regression models were used to assess multiple waves of data for each child. This was necessary as the data was clustered (i.e. multiple responses per child over time) and it is necessary to account for this non-independence of each observation. The models were adjusted for individual-level fixed effects which accounts for all time-invariant variation between individuals. This, for example, could be sex, genetic factors, unmeasured sociodemographic factors, early life determinants, or personality traits that could bias this relationship between transport and health. As these individual fixed effects were adjusted for, the models exploit the within-individual variation over time and measure the association between changes in travel mode and changes in adiposity whilst controlling for all (measured and unmeasured) time invariant characteristics. We additionally controlled for time-variant confounders in the models that could bias this association over time.

For peer review only

These models thus produce the average association between changing travel mode across all three-time points compared with changes among participants who used private transport.

To assess differences across our two markers of SEP we used interaction tests to assess if differences were statistically significantly different and present stratified analyses. All analyses used cluster robust standard errors to account for hetereoskeascity and autocorrelation, and employed survey weights to correct for differential response rates between groups and over time [20].

Exploratory analyses

In exploratory analyses we also investigated the potential that any differentials across SEP could be caused by differing duration of journeys to school, which may both determine mode of transport and be a proxy for amounts of physical activity involved in journeys. For these analyses we restricted to children walking or cycling to school and used the question *"On a typical day, how long does it take [^Cohort member's name] to get from home to school, one way*?" with options *"<5mins, 5-10 minutes, 15-30mins, 30-45mins, 45mins-1hour, 1-2 hours, 2 hours or more*". We have categorised this into a binary variable of 15 minutes or more vs. less than 15 minutes each way, as a proxy for journey distance. We conducted logistic regression to assess which participants were more likely to travel 15 minutes or more to school at age 14.

Results

Details of the sample and their characteristics at age 14 are given in **Table 1**. 26.1% of children used private motorised modes to travel to school, 35.3% used public transport and 38.6% used active modes. Levels of active travel ranged from 45.1% in the *Lower* NS-SEC groups to 35.2% among the M*anagerial/Professional* NS-SEC group, and were highest among those in the lowest income group (46.0%, versus 36.2% among those in the highest income households). The majority of children (5,409 64.1%) lived in England, were white (7,329, 86.9%) and had more than three hours per day of screen use (6,535, 77.5%). Modes of travel to school at previous ages are shown in **appendix tables 1 and 2.** These show for example, that 47.3% of children used private motorised modes to travel to school at age 7, compared with 43.7% at age 11.

Results from fixed effects longitudinal regression models are given in **Table 2**. BMI increased substantially between the ages of 7 and 14 years (+4.59 kg/m^2, Confidence Interval (CI) 4.45 to 4.73 for age 14 compared with age 7). Caregiver-reported beginning of growth spurts between ages 11 and 14 was associated with increased BMI (0.22 kg/m^2, 0.14 to 0.31). Switching to eating breakfast

For peer review only

every day was associated with lower BMI (-0.39 kg/m^2, -0.51 to -0.27), compared with not eating breakfast every day, as was moving to doing physical activity 5 days or more per week (-0.32 kg/m^2, -0.41 to -0.23). On average, after adjusting for these trends, switching from private to active travel was associated with a -0.21kg/m^2 lower BMI (95% Confidence Interval -0.31 to -0.10), while switching from private travel to public transport was associated with a -0.13 (-0.26 to 0.01) lower BMI.

Percentage body fat was higher at age 11 (+1.06%, 0.87 to 1.25) but not at age 14 (+0.21%, -0.14 to 0.56) than at age 7. Switching to eating breakfast seven days a week was associated with a -1.34% lower body fat (-1.62 to -1.06) but the effect of switching to 3 or more hours a day of screen use did not quite reach statistical significance (+0.22%, -0.01 to 0.44). Switching to 3 to 4 days a week of physical activity was associated with a -0.25% lower body fat (CI -0.48 to -0.01) and switching to 5 or more days a week of physical activity a -1.04% lower body fat (CI -1.25 to -0.82). Switching from a private mode to an active mode of travel was associated with a -0.55% lower percentage body fat (CI -0.80 to -0.31) and switching to public transport with a -0.43% lower percentage body fat CI (-0.75 to -0.12).

Associations of changes in travel mode with BMI across our markers of SEP are shown in **Figure 1** and **Appendix Table 3.** Interaction tests did not reveal differences between income groups (p=0.130) nor NS-SEC (p=0.074). However, visual interpretation of these charts did appear to show that these relationships were most concentrated in the lower SEP groups. For example, switching to active travel was associated with a -0.32kg/m^2 lower BMI (CI -0.58 to -0.06) among those in the lowest household income group compared with -0.11kg/m^2 among the highest household income group (CI -0.24 to 0.03). Similarly switching to active travel was associated with a -0.33kg/m^2 lower BMI (CI -0.55 to -0.11) among those in the *Not Economically Active* NS-SEC group, but a -0.09kg/m^2 lower BMI (CI -0.22, 0.04) among those in *Managerial/Professional* NS-SEC group.

Results were similar for percentage body fat and are shown in **Figure 2** and **Appendix Table 4**. Interaction tests did not reveal there to be statistically significant differences in associations between income groups (p=0.908) nor NS-SEC groups (p=0.128). Switching to active travel was associated with a -0.71% lower body fat (-1.47, 0.05) among children in the lowest household income group, compared with a -0.55% lower body fat (-1.01, -0.09) among those in the highest income group. Among the *Economically Inactive* NS-SEC group, switching to an active travel mode was associated with a -0.78% lower body fat (-1.27, -0.29) compared with a -0.43% lower body fat (-0.78, -0.08) among the *Managerial/Professional* NS-SEC group.

For peer review only

Exploratory analyses of the potential role of journey duration to school in findings by SEP found that more affluent children were more likely to travel more than 15 minutes each way to school. 60.5% of children in the M*anagerial/Professional* NS-SEC group travelled 15 minutes or further compared with 51.9% of those in the N*ot Economically Active* NS-SEC group (Adjusted Odds Ratio (AOR) 1.18, CI 1.04 to 1.34). 60.7% of children in the highest income households travelled 15 minutes or more to school compared with 48.6% of those in the lowest income households (AOR 1.21, 95%CI 1.03 to 1.42). Analyses interacting duration with mode of travel were restricted to only ages 11 and age 14 due to data availability, but interaction tests did not reveal a difference between the associations for walkers/cyclists more than vs. less than 15 minutes away for BMI (p= 0.259) nor body fat (p=0.452) (**Appendix Table 5**).

Discussion

This longitudinal study followed children over a key period in the lifecourse, and found that switching from private motorised modes of travel to school to active travel was associated with reduced objectively-measured adiposity. Additionally, switching to the use of public transport was associated with lower adiposity, although associations with BMI did not reach statistical significance. There was also a suggestion that these associations may be more concentrated among the most disadvantaged children, who are at greater risk of excess adiposity and relevant sequelae.

Strengths and limitations

This study used an established and representative UK birth cohort study with high quality data following up the same children over 8 years. We used two objective measures of adiposity and employed models that produce robust estimates of within-individual changes, which improves confidence in these results. We were able to adjust for unobserved time-invariant individual characteristics that can bias the relationship between travel mode and adiposity and are a major weakness of cross-sectional studies. Nonetheless, there are limitations to this research which should be considered. We rely on self-reported mode of travel to school, which while it was consistent across the study period, did not capture information on multi-mode journeys or variation in different modes across the week. This may have introduced some misclassification in our findings. Our reliance on these self-report questions is in contrast to some other research using the Millennium Cohort Study, which has used objective physical activity measures by accelerometers [21]. However, issues with using accelerometer data here include that is currently not available for the age 14

For peer review only

sweep; sample sizes being substantially lower for this method of data collection; and difficulties in ascertaining cycling to school. There was an average of three years between data collection points, and we do not know at what point between data collections individuals changed their travel behaviour. While we were able to consider a wide range of covariates, there may be some residual confounding from time-varying factors not considered [22]. There is also the potential for some confounding from other lifestyle factors as, for example, individuals who switch to more active modes may be choosing to lead a healthier lifestyle in other ways also, and changes in adiposity may be occurring via alternative mechanisms than active travel.

Comparison with other research and policy implications

This study contributes to the evidence base by using a robust longitudinal examination of changes in travel modes and adiposity as well as specifically investigating whether there are differences across markers of SEP. We use a national sample and we were able to employ more robust methods, control for more potential confounding factors and follow children up for longer compared to many previous studies. The overall findings add to the evidence base that active forms of travel to school are linked with more favourable levels of adiposity [10][23][24]. We also find no statistical evidence of differences between SEP groups, although there was a trend towards larger effects in more deprived groups. However, we caution against over interpretation of this finding given our restriction to only examining within-individual variation (hence reducing our statistical power) and the limited number of individuals within the study after stratifying by markers of SEP.

While we find higher levels of active travel use among more deprived groups, the wider evidence base on physical activity by markers of deprivation presents a mixed picture. Research using objective physical activity data from the MCS found that when they were aged seven, children in more deprived areas were more likely to be obese, but were also more likely to hit recommended levels of physical activity [25]. Recent reviews have questioned whether there are differences in physical activity by deprivation, and concluded that while more affluent children do more leisure time activity, overall differences remain small for physical activity overall and for active travel specifically [26]. We furthermore identified that switching to public transport was linked to reduced adiposity although associations with BMI did not reach statistical significance. There has been limited consideration of the impacts of public transportation use on health, but this finding does broadly concur with a recent systematic review in adults which identified 10 relevant studies. This

For peer review only

systematic review found that switching to public transport was associated with a -0.30 decrease in BMI, which is larger than our point estimate of -0.13, albeit in adults [27].

These findings highlight the potential for a shift to physically active modes of travel to play a role in reducing population level adiposity, and potentially to reduce health inequalities. They also reinforce calls from the World Health Organisation to develop policies which promote incorporating physical activity into daily lives [28]. However, there is mixed evidence on the how best to increase levels of active travel to school, which remains a fruitful avenue for future research [29]. Additionally, determinants of active travel are known to be wide ranging including parental concerns regarding safety, attitudes of the school and wider environmental factors [30][31][32][33][34]. Systematic reviews have found a wide range of potential determinants, including negative associations with traffic volumes, but that results were heterogeneous due to differing contexts and measures [35]. Nonetheless, active travel is only one potential mechanism to reduce adiposity in the population, and our findings also point to beneficial impacts of eating breakfast every day and sporting physical activity. For example, the associations identified here for switching to active forms of travel were considerably smaller than for switching to 5 or more days per week of physical activity for both BMI (-0.22kg/m^2 vs. -0.32kg/m^2) and body fat (-0.55% vs. -1.00%). Given the well-established benefits of physical activity on outcomes other than adiposity, and the potential additional benefits of increasing active travel in the form of air pollution and greenhouse gases, concerted action should be made to encourage the use of more active modes of travel to school [36].

Conclusion

This seven-year longitudinal study of children in the UK has concluded that switching to active forms of travel can have beneficial adiposity impacts at a key time in the lifecourse. These benefits may be more apparent among disadvantaged children. This research adds to the evidence base on the health impacts of active travel and suggests that targeted structural interventions may reduce inequalities in health in the UK.

For peer review only

Declarations

Patient and Public Involvement

Patients were not involved in the design of this study

Ethical approval

As this study involved secondary analysis the MCS ethical approval was not required. Data collection for the MCS has ethical approval from the Yorkshire and Humber ethics committee and further details are available from http://www.cls.ioe.ac.uk/.

Consent for publication

Not applicable

Availability of data and materials

The datasets generated during and/or analysed during the current study are available in the UKDataService repository https://ukdataservice.ac.uk/

Competing interests

All authors declare that they have no competing interests

Funding

This study/project is funded by the National Institute for Health Research (NIHR) School for Public Health Research (Grant Reference Number PD-SPH-2015) as well as a NIHR Research Professorship award to Professor Millett (NIHR RP_2014-04-032). The views expressed are those of the author(s) and not necessarily those of the NIHR or the Department of Health and Social Care

Authors' contributions

AL and CM conceived of the study. AL, CM and TH devised the analyses. AL conducted the analyses with the assistance of TH. AL produced the initial draft and all authors revised this for significant intellectual content and approve the final version

Acknowledgements

We would like to acknowledge the participants of the MCS

For peer review only

Table 1: Mode of travel to school in the Millennium Cohort Study in 2014/15, age 14/15 years

		N	Car	PT	Active
Gender	Male	4,102	24.2	35.4	40.4
	Female	4,330	27.9	35.2	36.9
Country	England	5,409	26.9	29.8	43.3
	Wales	1,182	25.5	40.3	34.2
	Scotland	958	20.0	40.5	39.5
	Northern Ireland	883	28.2	57.0	14.8
NS-SEC social class	Managerial/professional	2,653	27.5	37.2	35.2
	Intermediate	2,054	26.5	38.3	35.2
	Lower	1,710	22.9	32.0	45.1
	Not economically active	2,015	26.4	32.6	41.0
Household income group	Lowest	1,092	26.1	27.9	46.0
	Second lowest	1,286	23.2	36.5	40.4
	Middle	1,737	25.2	37.2	37.6
	Second highest	2,121	27.3	35.7	37.1
	Highest	2,196	27.3	36.5	36.2
Ethnic group	White	7,329	25.0	36.5	38.5
	Mixed	64	17.2	45.3	37.5
	Indian	208	34.6	26.4	38.9
	Pakistani or Bangladeshi	468	42.7	10.9	46.4
	Black or Black British	241	18.7	49.4	32.0
	Other	122	31.1	39.3	29.5
Growth spurt	Not yet/barely started	2,952	25.4	35.9	38.7
	Begun or completed	5,480	26.4	35.0	38.6
Breakfast consumption	< 7 days per week	3,824	25.8	35.0	39.2
	Every day	4,608	26.3	35.6	38.1
Portions of fruit per day	≤3 portions per day	5,775	26.1	34.5	39.3
	≥2 portions per day	2,655	25.9	37.1	37.0
Physical activity	≤2 days per week	2,342	27.8	34.3	37.9
	3-4 days per week	2,905	25.0	37.6	37.5
	≥ 5 days per week	3,185	25.8	34.0	40.2
TV or computer use	≤2 hours per day	1,897	26.4	38.2	35.4
	≥3 hours per day	6,535	26.0	34.5	39.5
Overall		8,432	26.1	35.3	38.6

PT = Public transport

For peer review only

Table 2: Results from longitudinal fixed-effects regression of impacts of switching school travel mode and adiposity

		BMI		% Body fat	
		coeff	95% CI	coeff	95% CI
Mode of travel	Private transport	ref	ref	ref	ref
	Public transport	-0.13	-0.26 ;0.01	-0.43	-0.75 ;-0.12
	Walk/Cycle	-0.21	-0.31 ;-0.10	-0.55	-0.80 ;-0.31
Age	7	ref	ref	ref	ref
	11	2.44	2.37 ;2.52	1.06	0.87 ;1.25
	14	4.59	4.45 ;4.73	0.21	-0.14 ;0.56
Country	England	ref	ref	ref	ref
	Wales	0.13	-1.10 ;1.37	1.77	-0.54 ;4.08
	Scotland	0.01	-1.00 ;1.01	0.46	-1.88 ;2.79
	Northern Ireland	-0.10	-1.92 ;1.72	1.07	-3.04 ;5.19
NS-SEC social class	Managerial/professional	ref	ref	ref	ref
	Self-employed intermediate	-0.16	-0.27 ;-0.05	-0.41	-0.71 ;-0.11
	Lower semi-routine routine	-0.12	-0.24 ;0.01	-0.37	-0.68 ;-0.06
	Not economically active	-0.08	-0.22 ;0.06	-0.16	-0.50 ;0.18
Household income group	Lowest	ref	ref	ref	ref
	Second lowest	0.08	-0.07 ;0.23	0.41	0.05 ;0.77
	Middle	0.13	-0.03 ;0.29	0.36	-0.02 ;0.75
	Second highest	0.06	-0.11 ;0.23	0.12	-0.30 ;0.54
	Highest	-0.01	-0.20 ;0.17	0.14	-0.30 ;0.59
Growth spurt	Not yet/barely started	ref	ref	ref	ref
	Begun or completed	0.22	0.14 ;0.31	0.1	-0.11 ;0.31
Breakfast consumption	< 7 days per week	ref	ref	ref	ref
	Every day	-0.39	-0.51 ;-0.27	-1.34	-1.62 ;-1.06
Portions of fruit per day	≤3 portions per day	ref	ref	ref	ref
	≥2 portions per day	0.05	-0.04 ;0.15	0.11	-0.11 ;0.33
Physical activity	≤2 days per week	ref	ref	ref	ref
	3-4 days per week	-0.06	-0.16 ;0.04	-0.25	-0.48 ;-0.01
	≥ 5 days per week	-0.32	-0.41 ;-0.23	-1.04	-1.25 ;-0.82
TV or computer use	≤2 hours per day	ref	ref	ref	ref
	≥3 hours per day	0.07	-0.02 ;0.16	0.22	-0.01 ;0.44

Models adjusted for individual-level fixed effects and changes in: country; highest NSSEC in household; household income; eating breakfast; frequency of eating breakfast; self-reported growth spurt; hours of TV and computer use per day; and days per week of moderate-to-vigorous physical activity.
Coeff = coefficient

For peer review only

Figure 1: Changes in BMI and 95% Confidence Intervals by mode of travel to school

Figure 2: Changes in % body fat and 95% Confidence Intervals by mode of travel to school

For peer review only

References

1 Ekelund U, Brage S, Froberg K, *et al.* TV viewing and physical activity are independently associated with metabolic risk in children: The European youth heart study. *PLoS Med* Published Online First: 2006. doi:10.1371/journal.pmed.0030488

2 Griffiths LJ, Cortina-Borja M, Sera F, *et al.* How active are our children? Findings from the millennium cohort study. *BMJ Open* Published Online First: 2013. doi:10.1136/bmjopen-2013-002893

3 Kwon S, Janz KF, Cooper A, *et al.* Tracking of accelerometry-measured physical activity during childhood: ICAD pooled analysis. *Int J Behav Nutr Phys Act* Published Online First: 2012. doi:10.1186/1479-5868-9-68

4 Hills AP, Andersen LB, Byrne NM. Physical activity and obesity in children. *Br J Sports Med* 2011;**45**:866 LP – 870. doi:10.1136/bjsports-2011-090199

5 Cooper AR, Goodman A, Page AS, *et al.* Objectively measured physical activity and sedentary time in youth: The International children's accelerometry database (ICAD). *Int J Behav Nutr Phys Act* Published Online First: 2015. doi:10.1186/s12966-015-0274-5

6 Morton KL, Corder K, Suhrcke M, *et al.* School polices, programmes and facilities, and objectively measured sedentary time, LPA and MVPA: Associations in secondary school and over the transition from primary to secondary school. *Int J Behav Nutr Phys Act* Published Online First: 2016. doi:10.1186/s12966-016-0378-6

7 Flint E, Cummins S, Sacker A. Associations between active commuting, body fat, and body mass index: population based, cross sectional study in the United Kingdom. *Br Med J* 2014;**349**. doi:10.1136/bmj.g4887

8 Roth MA, Millett CJ, Mindell JS. The contribution of active travel (walking and cycling) in children to overall physical activity levels: A national cross sectional study. *Prev Med (Baltim)* Published Online First: 2012. doi:10.1016/j.ypmed.2011.12.004

9 Colley R, Larouche R, Saunders TJ, *et al.* Associations between Active School Transport and Physical Activity, Body Composition, and Cardiovascular Fitness: A Systematic Review of 68 Studies. *J Phys Act Heal* Published Online First: 2016. doi:10.1123/jpah.2011-0345

10 Lubans DR, Boreham CA, Kelly P, *et al.* The relationship between active travel to school and health-related fitness in children and adolescents: A systematic review. Int. J. Behav. Nutr. Phys. Act. 2011. doi:10.1186/1479-5868-8-5

11 Department for Transport (2015). National Travel Survey 2014: Travel to school https://assets.publishing.service.gov.uk/government/uploads/system/uploads/attachment_data/file/476635/travel-to-school.pdf.

12 Pouliou T, Sera F, Griffiths L, *et al.* Environmental influences on children's physical activity. *J Epidemiol Community Health* Published Online First: 2015. doi:10.1136/jech-2014-204287

13 Laverty AA, Mindell JS, Webb EA, *et al.* Active travel to work and cardiovascular risk factors in the united kingdom. *Am J Prev Med* 2013;**45**:282–8. doi:10.1016/j.amepre.2013.04.012

14 Higgins, V., Dale, A., 2010. Ethnic differences in physical activity and obesity, working paper. Cathie Marsh Centre for Census and Survey Research https://lemosandcrane.co.uk/resources/University%20of%20Manchester%20-%20Ethnic%20differences%20in%20physic.

15 Cavill N, Davis A, Cope A, Corner D, 2019, Active Travel and Physical Activity Evidence Review. Sport England https://www.sportengland.org/media/13943/active-travel-full-report-evidence-review.pdf.

16 Su JG, Jerrett M, McConnell R, *et al.* Factors influencing whether children walk to school. *Health Place* 2013;**22**:153–61. doi:https://doi.org/10.1016/j.healthplace.2013.03.011

17 Noonan RJ, Boddy LM, Knowles ZR, *et al.* Fitness, fatness and active school commuting among liverpool schoolchildren. *Int J Environ Res Public Health* Published Online First: 2017. doi:10.3390/ijerph14090995

18 Schoeppe S, Duncan MJ, Badland H, *et al.* Associations of children's independent mobility and

active travel with physical activity, sedentary behaviour and weight status: A systematic review. *J Sci Med Sport* 2013;**16**:312–9. doi:https://doi.org/10.1016/j.jsams.2012.11.001

19 Saunders LE, Green JM, Petticrew MP, *et al.* What Are the Health Benefits of Active Travel? A Systematic Review of Trials and Cohort Studies. *PLoS One* 2013;**8**:e69912. doi:10.1371/journal.pone.0069912

20 Connelly R, Platt L. Cohort profile: UK Millennium Cohort Study (mcs). *Int J Epidemiol* 2014;**43**:1719–25. doi:10.1093/ije/dyu001

21 Griffiths LJ, Sera F, Cortina-Borja M, *et al.* Objectively measured physical activity and sedentary time: cross-sectional and prospective associations with adiposity in the Millennium Cohort Study. *BMJ Open* 2016;**6**:e010366. doi:10.1136/bmjopen-2015-010366

22 Harrison F, Goodman A, van Sluijs EMF, *et al.* Weather and children's physical activity; how and why do relationships vary between countries? *Int J Behav Nutr Phys Act* 2017;**14**:74. doi:10.1186/s12966-017-0526-7

23 Schoeppe S, Duncan MJ, Badland H, *et al.* Associations of children's independent mobility and active travel with physical activity, sedentary behaviour and weight status: A systematic review. *J Sci Med Sport* 2013;**16**:312–9. doi:https://doi.org/10.1016/j.jsams.2012.11.001

24 Faulkner GEJ, Buliung RN, Flora PK, *et al.* Active school transport, physical activity levels and body weight of children and youth: A systematic review. *Prev Med (Baltim)* 2009;**48**:3–8. doi:http://dx.doi.org/10.1016/j.ypmed.2008.10.017

25 Noonan RJ, Fairclough SJ. Is there a deprivation and maternal education gradient to child obesity and moderate-to-vigorous physical activity? Findings from the Millennium Cohort Study. *Pediatr Obes* 2018;**13**:458–64. doi:10.1111/ijpo.12287

26 Stalsberg R, Pedersen AV. Are Differences in Physical Activity across Socioeconomic Groups Associated with Choice of Physical Activity Variables to Report? *Int J Environ Res Public Health* 2018;**15**:922. doi:10.3390/ijerph15050922

27 Patterson R, Webb E, Hone T, *et al.* Associations of Public Transportation Use With Cardiometabolic Health: A Systematic Review and Meta-Analysis. *Am J Epidemiol* 2019;**188**:785–95. doi:10.1093/aje/kwz012

28 World Health Organization. Global action plan for the prevention and control of NCDs 2013-2020. Published Online First: 2013.http://www.who.int/nmh/publications/ncd-action-plan/en/

29 Larouche R, Mammen G, Rowe DA, *et al.* Effectiveness of active school transport interventions: a systematic review and update. *BMC Public Health* 2018;**18**:206. doi:10.1186/s12889-017-5005-1

30 Garnham-Lee KP, Falconer CL, Sherar LB, *et al.* Evidence of moderation effects in predicting active transport to school. *J Public Health (Oxf)* 2017;**39**:153–62. doi:10.1093/pubmed/fdw016

31 Panter J, Corder K, Griffin SJ, *et al.* Individual, socio-cultural and environmental predictors of uptake and maintenance of active commuting in children: longitudinal results from the SPEEDY study. *Int J Behav Nutr Phys Act* 2013;**10**:83. doi:10.1186/1479-5868-10-83

32 Carver A, Salmon J, Campbell K, *et al.* How Do Perceptions of Local Neighborhood Relate to Adolescents' Walking and Cycling? *Am J Heal Promot* 2005;**20**:139–47. doi:10.4278/0890-1171-20.2.139

33 Panter JR, Jones AP, van Sluijs EMF, *et al.* Attitudes, social support and environmental perceptions as predictors of active commuting behaviour in school children. *J Epidemiol Community Health* 2010;**64**:41–8. doi:10.1136/jech.2009.086918

34 Morton KL, Atkin AJ, Corder K, *et al.* The school environment and adolescent physical activity and sedentary behaviour: a mixed-studies systematic review. *Obes Rev* 2016;**17**:142–58. doi:10.1111/obr.12352

35 Sharmin S, Kamruzzaman M. Association between the built environment and children's independent mobility: A meta-analytic review. *J Transp Geogr* 2017;**61**:104–17.

For peer review only

doi:https://doi.org/10.1016/j.jtrangeo.2017.04.004
36 Woodcock J, Edwards P, Tonne C, *et al.* Public health benefits of strategies to reduce greenhouse-gas emissions: urban land transport. *Lancet* 2009;**374**:1930–43. doi:S0140-6736(09)61714-1 [pii] 10.1016/S0140-6736(09)61714-1

For peer review only

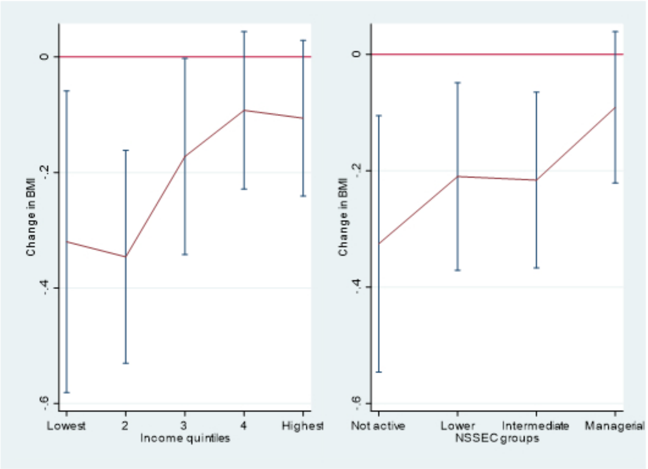

Figure 1: Changes in BMI and 95% Confidence Intervals by mode of travel to school

216x121mm (96 x 96 DPI)

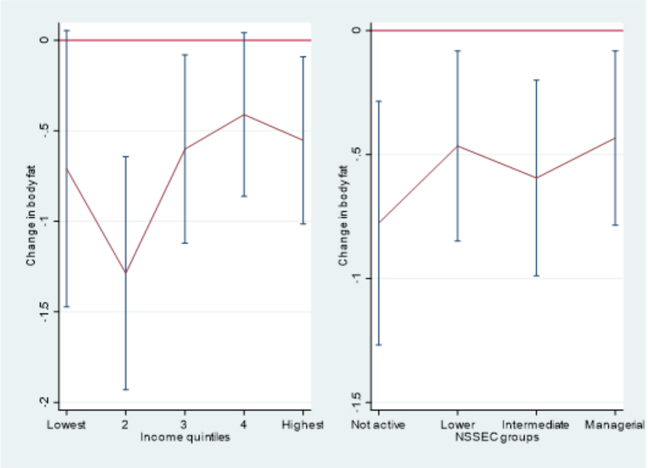

Figure 2: Changes in % body fat and 95% Confidence Intervals by mode of travel to school

216x121mm (96 x 96 DPI)

Appendix table 1: Mode of travel to school in the Millennium Cohort Study in 2011/12, age 10-11 years

		Car	PT	Active
Gender	Male	47.4	5.3	47.3
	Female	47.2	5.2	47.6
Country	England	45.5	2.6	51.9
	Wales	51.1	7.6	41.3
	Scotland	37.8	9.8	52.4
	Northern Ireland	62.9	13.9	23.1
NS-SEC social class	Not economically active	28.7	4.9	66.4
	Lower	39.7	5.7	54.6
	Intermediate	53.7	4.9	41.4
	Managerial/professional	53.7	5.1	41.2
Household income group	Lowest	34.6	5.5	59.9
	Second lowest	43.3	6.6	50.0
	Middle	47.8	5.2	47.0
	Second highest	51.1	4.5	44.4
	Highest	56.2	4.7	39.1
Ethnic group	White	48.0	5.7	46.3
	Mixed	41.6	4.7	53.7
	Indian	58.4	1.0	40.6
	Pakistani or Bangladeshi	38.1	1.1	60.8
	Black or Black British	40.5	6.2	53.3
	Other	43.5	3.3	53.3
Growth spurt*	Not yet/barely started	-	-	-
	Begun or completed	-	-	-
Breakfast consumption	< 7 days per week	42.9	7.3	49.8
	Every day	47.6	5.1	47.3
Portions of fruit per day	≤3 portions per day	46.1	5.1	48.8
	≥2 portions per day	47.6	5.3	47.1
Physical activity	≤2 days per week	48.9	4.2	46.9
	3-4 days per week	50.1	5.0	44.9
	≥ 5 days per week	46.2	5.7	48.1
TV or computer use	≤2 hours per day	47.5	5.5	47.0
	≥3 hours per day	46.4	4.2	49.4
Overall		47.3	5.3	47.4

PT = Public Transport , * N/A

For peer review only

Appendix table 2: Mode of travel to school in the Millennium Cohort Study in 2007/08, age 7-8 years

		Car	PT	Active
Gender	Male	42.6	6.6	50.9
	Female	44.7	6.9	48.4
Country	England	42.1	4.7	53.2
	Wales	47.1	8.4	44.5
	Scotland	32.2	10.1	57.8
	Northern Ireland	60.8	13.6	25.6
NS-SEC social class	Not economically active	36.8	6.8	56.4
	Lower	38.3	5.9	55.7
	Intermediate	47.8	6.4	45.8
	Managerial/professional	50.7	7.4	41.8
Household income group	Lowest	31.0	6.4	62.6
	Second lowest	40.2	8.2	51.7
	Middle	48.5	6.8	44.7
	Second highest	48.7	6.0	45.3
	Highest	46.3	6.4	47.3
Ethnic group	White	43.9	6.9	49.2
	Mixed	42.2	10.1	47.7
	Indian	55.3	2.6	42.1
	Pakistani or Bangladeshi	41.3	1.8	56.9
	Black or Black British	33.6	12.3	54.0
	Other	40.0	5.6	54.4
Growth spurt	Not yet/barely started	45.0	6.5	48.5
	Begun or completed	42.3	7.0	50.7
Breakfast consumption	< 7 days per week	40.0	8.5	51.5
	Every day	44.2	6.5	49.3
Portions of fruit per day	≤3 portions per day	42.3	7.3	50.5
	≥2 portions per day	44.3	6.5	49.2
Physical activity	≤2 days per week	44.7	6.8	48.5
	3-4 days per week	44.4	6.6	49.0
	≥ 5 days per week	42.7	6.7	50.6
TV or computer use	≤2 hours per day	44.5	7.1	48.4
	≥3 hours per day	39.9	5.0	55.1
Overall		43.7	6.7	49.6

For peer review only

Appendix Table 3: Results from longitudinal fixed-effects regression of impacts of switching school travel mode and BMI stratified by markers of Socio-Economic Position

NSSEC groups	Coefficient	Lower CI	Upper CI
Managerial/professional	-0.09	-0.22	0.04
Self-employed intermediate	-0.22	-0.37	-0.07
Lower semi-routine routine	-0.21	-0.37	-0.05
Not economically active	-0.33	-0.55	-0.11
Household income groups			
Highest quantile income	-0.11	-0.24	0.03
Fourth quantile	-0.09	-0.23	0.04
Third quantile	-0.17	-0.34	0.00
Second quantile	-0.35	-0.53	-0.16
Lower quantile income	-0.32	-0.58	-0.06

NSSEC = National Statistics Socio-economic Classification, CI = Confidence Interval

Models adjusted for individual-level fixed effects and changes in: country; highest NSSEC in household; household income; eating breakfast; frequency of eating breakfast; self-reported growth spurt; hours of TV and computer use per day; and days per week of moderate-to-vigorous physical activity.

Appendix Table 4: Results from longitudinal fixed-effects regression of impacts of switching school travel mode and percentage body fat stratified by markers of Socio-Economic Position

NSSEC groups	Coefficient	Lower CI	Upper CI
Managerial/professional	-0.43	-0.78	-0.08
Self-employed intermediate	-0.59	-0.99	-0.20
Lower semi-routine routine	-0.47	-0.85	-0.08
Not economically active	-0.78	-1.27	-0.29
Household income groups			
Highest quantile income	-0.55	-1.01	-0.09
Fourth quantile	-0.41	-0.86	0.04
Third quantile	-0.60	-1.12	-0.08
Second quantile	-1.29	-1.93	-0.64
Lower quantile income	-0.71	-1.47	0.05

Models adjusted for individual-level fixed effects and changes in: country; highest NSSEC in household; household income; eating breakfast; frequency of eating breakfast; self-reported growth spurt; hours of TV and computer use per day; and days per week of moderate-to-vigorous physical activity.

For peer review only

Appendix table 5: Fully adjusted odds ratios travelling 15 minutes or more each way by walking or cycling

		%	OR	Lower CI	Upper CI
Gender	Male	42.2	ref	ref	ref
	Female	45.1	1.14	1.00	1.30
Country	England	45.2	ref	ref	ref
	Wales	38.5	0.65	0.53	0.79
	Scotland	41.2	0.74	0.61	0.91
	Northern Ireland	36.6	0.60	0.43	0.83
NS-SEC social class	Not economically active	39.7	ref	ref	ref
	Lower	43.8	1.10	0.91	1.33
	Intermediate	44.5	1.07	0.87	1.32
	Managerial/professional	47.0	1.20	0.98	1.47
Household income group	Lowest	38.2	ref	ref	ref
	Second lowest	43.8	1.00	0.80	1.25
	Middle	44.9	0.98	0.77	1.23
	Second highest	44.9	0.88	0.69	1.11
	Highest	46.0	0.86	0.66	1.11
Ethnic group	White	46.2	ref	ref	ref
	Mixed	53.8	1.30	0.67	2.53
	Indian	36.4	0.60	0.41	0.88
	Pakistani or Bangladeshi	23.9	0.30	0.23	0.40
	Black or Black British	38.9	0.66	0.45	0.97
	Other	32.3	0.53	0.31	0.90
Growth spurt	Not yet/barely started	43.3	ref	ref	ref
	Begun or completed	44.2	0.99	0.87	1.13
Breakfast consumption	< 7 days per week	43.3	ref	ref	ref
	Every day	44.0	1.07	0.94	1.22
Portions of fruit per day	≤3 portions per day	43.4	ref	ref	ref
	≥2 portions per day	44.4	1.00	0.87	1.16
Physical activity	≤2 days per week	42.7	ref	ref	ref
	3-4 days per week	45.6	1.14	0.97	1.34
	≥ 5 days per week	42.5	0.98	0.83	1.15
TV or computer use	≤2 hours per day	42.5	ref	ref	ref
	≥3 hours per day	43.9	1.01	0.86	1.18

For peer review only

STROBE 2007 (v4) Statement—Checklist of items that should be included in reports of *cohort studies*

Section/Topic	Item #	Recommendation	Reported on page #
**Title and abstract**	1	(*a*) Indicate the study's design with a commonly used term in the title or the abstract	1
		(*b*) Provide in the abstract an informative and balanced summary of what was done and what was found	1 - 2
**Introduction**			
Background/rationale	2	Explain the scientific background and rationale for the investigation being reported	4 - 5
Objectives	3	State specific objectives, including any prespecified hypotheses	5
**Methods**			
Study design	4	Present key elements of study design early in the paper	4 - 5
Setting	5	Describe the setting, locations, and relevant dates, including periods of recruitment, exposure, follow-up, and data collection	5 - 6
Participants	6	(*a*) Give the eligibility criteria, and the sources and methods of selection of participants. Describe methods of follow-up	5 - 6
		(*b*) For matched studies, give matching criteria and number of exposed and unexposed	n / a
Variables	7	Clearly define all outcomes, exposures, predictors, potential confounders, and effect modifiers. Give diagnostic criteria, if applicable	5 - 7
Data sources/ measurement	8*	For each variable of interest, give sources of data and details of methods of assessment (measurement). Describe comparability of assessment methods if there is more than one group	*6*
Bias	9	Describe any efforts to address potential sources of bias	6
Study size	10	Explain how the study size was arrived at	5
Quantitative variables	11	Explain how quantitative variables were handled in the analyses. If applicable, describe which groupings were chosen and why	5 - 6
Statistical methods	12	(*a*) Describe all statistical methods, including those used to control for confounding	5 – 6
		(*b*) Describe any methods used to examine subgroups and interactions	6
		(*c*) Explain how missing data were addressed	6
		(*d*) If applicable, explain how loss to follow-up was addressed	6
		(*e*) Describe any sensitivity analyses	6
**Results**			

For peer review only

Participants	13*	(a) Report numbers of individuals at each stage of study—eg numbers potentially eligible, examined for eligibility, confirmed eligible, included in the study, completing follow-up, and analysed	6
		(b) Give reasons for non-participation at each stage	5 – 6
		(c) Consider use of a flow diagram	n / a
Descriptive data	14*	(a) Give characteristics of study participants (eg demographic, clinical, social) and information on exposures and potential confounders	Table 1
		(b) Indicate number of participants with missing data for each variable of interest	Table 1
		(c) Summarise follow-up time (eg, average and total amount)	5 - 6
Outcome data	15*	Report numbers of outcome events or summary measures over time	7
Main results	16	(*a*) Give unadjusted estimates and, if applicable, confounder-adjusted estimates and their precision (eg, 95% confidence interval). Make clear which confounders were adjusted for and why they were included	7
		(*b*) Report category boundaries when continuous variables were categorized	7 – 9
		(*c*) If relevant, consider translating estimates of relative risk into absolute risk for a meaningful time period	8
Other analyses	17	Report other analyses done—eg analyses of subgroups and interactions, and sensitivity analyses	8
**Discussion**			
Key results	18	Summarise key results with reference to study objectives	9
**Limitations**			
Interpretation	20	Give a cautious overall interpretation of results considering objectives, limitations, multiplicity of analyses, results from similar studies, and other relevant evidence	9
Generalisability	21	Discuss the generalisability (external validity) of the study results	10
**Other information**			
Funding	22	Give the source of funding and the role of the funders for the present study and, if applicable, for the original study on which the present article is based	12

*Give information separately for cases and controls in case-control studies and, if applicable, for exposed and unexposed groups in cohort and cross-sectional studies.

Note: An Explanation and Elaboration article discusses each checklist item and gives methodological background and published examples of transparent reporting. The STROBE checklist is best used in conjunction with this article (freely available on the Web sites of PLoS Medicine at http://www.plosmedicine.org/, Annals of Internal Medicine at http://www.annals.org/, and Epidemiology at http://www.epidem.com/). Information on the STROBE Initiative is available at www.strobe-statement.org.

For peer review only

BMJ Open

Associations of active travel with adiposity among children and socio-economic differentials: a longitudinal study

Journal:	*BMJ Open*
Manuscript ID	bmjopen-2019-036041.R1
Article Type:	Original research
Date Submitted by the Author:	05-Oct-2020

Complete List of Authors:	Laverty, Anthony; Imperial College London<br>Hone, Thomas; Imperial College London,<br>Goodman, Anna; LSHTM, Faculty of Epidemiology and Population Health<br>Kelly, Yvonne; University College London, Epidemiology and Public Health<br>Millett, Christopher; Imperial College London

**Primary Subject Heading**:	Public health
Secondary Subject Heading:	Health policy
Keywords:	PUBLIC HEALTH, Community child health < PAEDIATRICS, PREVENTIVE MEDICINE

SCHOLARONE™
Manuscripts

I, the Submitting Author has the right to grant and does grant on behalf of all authors of the Work (as defined in the below author licence), an exclusive licence and/or a non-exclusive licence for contributions from authors who are: i) UK Crown employees; ii) where BMJ has agreed a CC-BY licence shall apply, and/or iii) in accordance with the terms applicable for US Federal Government officers or employees acting as part of their official duties; on a worldwide, perpetual, irrevocable, royalty-free basis to BMJ Publishing Group Ltd ("BMJ") its licensees and where the relevant Journal is co-owned by BMJ to the co-owners of the Journal, to publish the Work in this journal and any other BMJ products and to exploit all rights, as set out in our licence.

The Submitting Author accepts and understands that any supply made under these terms is made by BMJ to the Submitting Author unless you are acting as an employee on behalf of your employer or a postgraduate student of an affiliated institution which is paying any applicable article publishing charge ("APC") for Open Access articles. Where the Submitting Author wishes to make the Work available on an Open Access basis (and intends to pay the relevant APC), the terms of reuse of such Open Access shall be governed by a Creative Commons licence – details of these licences and which Creative Commons licence will apply to this Work are set out in our licence referred to above.

Other than as permitted in any relevant BMJ Author's Self Archiving Policies, I confirm this Work has not been accepted for publication elsewhere, is not being considered for publication elsewhere and does not duplicate material already published. I confirm all authors consent to publication of this Work and authorise the granting of this licence.

For peer review only

Associations of active travel with adiposity among children and socio-economic differentials: a longitudinal study

Anthony A. Laverty[1*], Thomas Hone[1], Anna Goodman[2], Yvonne Kelly[3], Christopher Millett[1]

[1] Public Health Policy Evaluation Unit, School of Public Health, Imperial College London

[2] Faculty of Epidemiology and Population Health, London School of Hygiene and Tropical Medicine

[3] Department of Epidemiology and Public Health, 1-19 Torrington Place, University College London, London, WC1E 6BT, UK

* Corresponding author a.laverty@imperial.ac.uk

For peer review only

Objectives

Examine longitudinal associations between modes of travel to school and adiposity

Setting

UK

Participants

8,432 children surveyed at ages 7, 11 and 14 years from the UK Millennium Cohort study.

Primary and secondary outcomes

Objective percentage body fat and Body Mass Index (BMI). Transport mode was categorised as private motorised transport, public transport, active transport (walking or cycling). Socio-Economic Position (SEP) was measured by household income group and occupational social class We adjusted analyses for changes in country of United Kingdom, frequency of eating breakfast, self-reported growth spurts, *hours of screen time, and days per week of moderate-to-vigorous physical activity.* Longitudinal (panel) regression models adjusting for individual fixed effects examined associations in changes in mode of travel to school and adiposity, controlling for both time-varying and time-invariant potential confounders. Interaction tests and stratified analyses investigated differences by markers of SEP.

Results

At age 14, 26.1% of children (2,198) reported using private motorised transport, 35.3% (2979) used public transport, and 38.6% (3,255) used active transport to get to school. 36.6% (3,083) of children changed mode twice between the three waves and 50.7% (4,279) changed once. Compared with continuing to use private transport, switching to active transport was associated with a lower BMI (-0.21kg/m^2, 95% Confidence Interval -0.31;-0.10) and body fat (-0.55%, -0.80;-0.31). Switching to public transport was associated with lower percentage body fat (-0.43%, -0.75;-0.12), but associations with BMI did not reach statistical significance (-0.13kg/m2, -0.26; 0.01). Interaction tests showed a trend for these effects to be stronger in more deprived groups, but these interactions did not reach statistical significance

Conclusion

This longitudinal study during a key lifecourse period found switching to physically active forms of travel can have beneficial adiposity impacts; these associations may be more apparent for more disadvantaged children. Increasing active travel has potential to ameliorate inequalities.

For peer review only

Strengths and limitations of this study

- Used an established and representative UK birth cohort study with high quality data following up the same children over 8 years.
- We used two objective measures of adiposity and employed models that produce robust estimates of within-individual changes and were able to adjust for unobserved time-invariant individual characteristics
- However, we rely on self-reported mode of travel to school, which did not capture information on multi-mode journeys or variation in different modes across the week.
- There was an average of three years between data collection points, and we do not know at what point between data collections individuals changed their travel behaviour.
- While we were able to consider a wide range of covariates, there may be some residual confounding from time-varying factors not considered such as choosing to lead a healthier lifestyle in other ways also, and changes in adiposity may be occurring via alternative mechanisms than active travel.

For peer review only

Introduction

Insufficient physical activity is associated with increased cardio-metabolic risk, including higher levels of adiposity in children as well as adults [1]. Children are an important group for physical activity promotion and the majority of children and adolescents do not reach recommended levels [2][3]. Additionally evidence from both the UK and international studies suggests that levels of physical activity track across the lifecourse, meaning that those who are insufficiently active early in life are at risk of this continuing into later life [4][5][6]. The period of transition from primary to secondary education is characterised by significant changes to children's social and physical environment which can strongly influence health behaviours [7].

Active travel (that is, the use of non-motorised modes of travel) has the potential to increase physical activity and reduce adiposity [8]. The use of active travel in children has been linked to higher levels of physical activity in a range of settings, including during the journey to school [6][9][10][11]. Modes of travel change significantly during childhood, with the majority of travel being with caregivers in primary school and then changing to more independent modes later [12]. There are also significant differentials across socio-economic position (SEP) in physical activity profiles, use of active travel, and cardio-metabolic health risks in children, which together contribute to levels of health inequalities between groups [13][14][15]. Inequalities are also present among children for other factors and behaviours which are associated with greater risks of obesity and ill-health, including screen time and dietary behaviours [16].While active travel may have the potential to offset some of these inequalities, a recent review by Sport England concluded that there is a comparative lack of evidence on the impacts of active travel across groups across markers of SEP [17].

Much of the evidence on the impacts of active travel has been cross-sectional and there has been a lack of evidence using large-scale prospective studies, especially in children. Studies have highlighted conflicting evidence on the potential role of active travel to school on body composition and adiposity and, a recent systematic review found a limited number of prospective studies in children and did not identify an association between active travel and obesity in children [18][19][20][21]. This study adds to the evidence base by using a robust analytical strategy on data from a representative cohort of children during a key period in their development to investigate the role of active travel to school on objectively measured adiposity, and the potential for differential effects according to two different measures of SEP. Multivariate longitudinal (panel) regression models are employed to follow the same individuals over time, adjust for time invariant factors, and assess changes in transport usage and health outcomes.

For peer review only

Methods

Sample and data

Data for this study came from the Millennium Cohort Study (MCS), which is a birth cohort study in the UK of children born between September 2000 and January 2002, sampled at approximately 9 months old [22]. Children were initially sampled in the year 2000 and follow up data is currently available from six sweeps. Sampling was based on a cluster-stratified framework with smaller population groups being over sampled, including those in the smaller nations of the UK, as well as those in disadvantaged areas and from ethnic minority backgrounds.

These analyses use data from the fourth sweep of data collection when children were aged on average 7years old (response rate 82% and age range 6-8), the fifth sweep of data collection children were on average11 years old (response rate 81%, and age range 10-12), and the sixth sweep of data when aged on average 14years old (response rate 76%, age range 13-15). A range of topics covering the economic, social and health circumstances of children are covered alongside objective adiposity measurements. We focused on these sweeps of data as levels of active travel were low before the age of 7 years. Questionnaires and objective measures are administered by trained interviewers to standardised protocols and further details are available from http://www.cls.ioe.ac.uk/ [22]. Specific ethical approval was not required for this study as it involved only the secondary analyses of data that does not contain identifiable information and participants give consent for their data to be used for research purposes

Variables

This study used two objective measures of adiposity at all three time points: Body Mass Index (BMI) and percentage body fat. Height was measured using a Leicester stadiometer, and weight and body fat were measured using Tanita BF-522 W scales [22].

The exposure for this study was mode of travel to school assessed by asking caregivers "*How does (child) usually travel to school?",* with prompts to give the mode with longest distance if more than one mode was used. There were seven potential responses: Public transport; School or local authority bus; minibus or coach; Car or other vehicle (including Taxi); Bicycle; Walking; and Other. We categorised these responses into three categories of private motorised (Car or other vehicle (including Taxi)), public transport (Public transport; School or local authority bus; minibus or coach) and active travel (walking and cycling). We combined walking and cycling into one overall category due to low numbers of cycling: 76 at age 7, 187 at age 11 and 147 at age 14. We excluded "Other" responses (253 at age 14 (2.2%), 1,203 at age 11 (9.0%), 174 (1.3% at age 7)) due to uncertainty

For peer review only

about what modes of travel these may be and the fact that they likely represent a heterogeneous mix of modes.

A range of covariates were included as potential confounders of the relationship between travel mode and adiposity, covering socioeconomic factors and health behaviours. Included covariates from all ages were sex; country of residence (England, Wales, Northern Ireland, Scotland); self-reported ethnicity; portions of fruit consumed per day (<3 vs. ≥3 pieces per day); breakfast consumption (seven days per week vs. not); screen use (hours per weekday watching TV or using a computer categorised as <3hours vs. ≥ 3 hours). We also created a variable to reflect days per week of sport/exercise each week (categorised as < once a week, 1–2 times a week, 3+ times a week). We also used data on whether caregivers report that their child has experienced a growth spurt, assessed using the question "*Most children have a growth spurt as they approach and during their teens. Would you say that [Cohort child's name]'s growth spurt ....?"* This question was asked only at ages 11 and 14 and we categorised this into two categories of has definitely started vs. not.

We used two markers of SEP both ascertained by self-report: household income group (equivalised based on the Organisation for Economic Co-operation and Development and categorised in five groups) and household occupational social class measured using the National Statistics Socio-Economic Classification (NS-SEC; categorised as *Not economically active; Lower; Intermediate; Managerial/Professional*).

For longitudinal analyses we included children who had complete data available from ages 7 to 14. For cross-sectional analyses at age 14 there were 11,714 children in the sample, of which we used data from 10,722 (91.5%) after excluding 253 (2.2%) for using an "other" form of transport and 739 (6.3%) for missing data on relevant covariates. For longitudinal analyses we include data from 8,432 children (82.5% of those with complete data at age 14) who had data on all relevant data from ages 7 to 14.

Analyses

The distribution of modes of travel to school across socio-demographic and behavioural characteristics was first examined descriptively.

Multivariate longitudinal (panel) regression models were used to assess multiple waves of data for each child. These models allow individuals to be followed over time, and also account for the

For peer review only

clustered nature of the data (i.e. multiple responses per child over time)[23]. Linear specifications were chosen due to the continuous nature of the variables of interest. Fixed effects specifications where chosen to adjust for individual time-invariant characteristics of individuals. These for example, could be sex, genetic factors, unmeasured sociodemographic factors, early life determinants, or personality traits that could bias this relationship between transport and health. As these individual fixed effects were adjusted for, the models exploit the within-individual variation over time and measure the association between changes in travel mode and changes in adiposity whilst controlling for all (measured and unmeasured) time invariant characteristics[24][23]. We additionally controlled for time-variant confounders in the models that could bias this association over time. This we controlled for changes in *country; highest NSSEC in household; household income; eating breakfast; frequency of eating breakfast; self-reported growth spurt; hours of TV and computer use per day; and days per week of moderate-to-vigorous physical activity.* Additionally time fixed effects were adjusted for with dummies for each wave of data to account for changes over time. The reported effect sizes from these models are interpreted as the the average association between changing travel mode across all three-time points compared with changes among participants who used private transport.

First, these models were used in two separate models to assess the associations between active transport and BMI and percentage body fat. Second to assess differences across our two markers of SEP we used interaction tests to assess if differences were statistically significantly different and present stratified analyses. All analyses used cluster robust standard errors to account for hetereoskeascity and autocorrelation, and employed survey weights to correct for differential response rates between groups and over time [22][23]. Analyses were conducted using Stata 14.0 and we implemented using the `xtreg` package for longitudinal analysis.

Exploratory analyses

In exploratory analyses we also investigated the potential that any differentials across SEP could be caused by differing duration of journeys to school, which may both determine mode of transport and be a proxy for amounts of physical activity involved in journeys. We restricted these analyses to individuals using physically active travel modes (walking or cycling to school). We used the question *"On a typical day, how long does it take [^Cohort member's name] to get from home to school, one way*?" with options *"<5mins, 5-10 minutes, 15-30mins, 30-45mins, 45mins-1hour, 1-2 hours, 2 hours or more*". We have categorised this into a binary variable of 15 minutes or more vs. less than 15 minutes each way, as a proxy for journey distance. We conducted logistic regression to assess which participants were more likely to travel 15 minutes or more to school at age 14.

For peer review only

Patient and Public Involvement

Patients were not involved in the design of this study

Results

Details of the sample and their characteristics at age 14 are given in **Table 1**. 26.1% of children used private motorised modes to travel to school, 35.3% used public transport and 38.6% used active modes. Levels of active travel ranged from 45.1% in the *Lower* NS-SEC groups to 35.2% among the M*anagerial/Professional* NS-SEC group, and were highest among those in the lowest income group (46.0%, versus 36.2% among those in the highest income households). The majority of children (5,409 64.1%) lived in England, were white (7,329, 86.9%) and had more than three hours per day of screen use (6,535, 77.5%). Modes of travel to school at previous ages are shown in **appendix tables 1 and 2.** These show for example, that 47.3% of children used private motorised modes to travel to school at age 7, compared with 43.7% at age 11. Levels of change in transport mode were high: 36.6% (3,083) of children changed mode twice between the three waves; 50.7% (4,279) changed once, and 12.7% (1,070) did not change travel mode. Overall switching between modes was more common between ages 11 and 14 (4,340 participants (51.5% of sample) changed travel mode), than between ages 7 and 11 (2,079 participants (24.7% of sample).

Results from fixed effects longitudinal regression models are given in **Table 2**. BMI increased substantially between the ages of 7 and 14 years (+4.59 kg/m^2, Confidence Interval (CI) 4.45 to 4.73 for age 14 compared with age 7). Caregiver-reported beginning of growth spurts between ages 11 and 14 was associated with increased BMI (0.22 kg/m^2, 0.14 to 0.31). Switching to eating breakfast every day was associated with lower BMI (-0.39 kg/m^2, -0.51 to -0.27), compared with not eating breakfast every day, as was moving to doing physical activity 5 days or more per week (-0.32 kg/m^2, -0.41 to -0.23). On average, after adjusting for these trends, switching from private to active travel was associated with a -0.21kg/m^2 lower BMI (95% Confidence Interval -0.31 to -0.10), while switching from private travel to public transport was associated with a -0.13 (-0.26 to 0.01) lower BMI.

Percentage body fat was higher at age 11 (+1.06%, 0.87 to 1.25) but not at age 14 (+0.21%, -0.14 to 0.56) than at age 7. Switching to eating breakfast seven days a week was associated with a -1.34%

lower body fat (-1.62 to -1.06) but the effect of switching to 3 or more hours a day of screen use did not quite reach statistical significance (+0.22%, -0.01 to 0.44). Switching to 3 to 4 days a week of physical activity was associated with a -0.25% lower body fat (CI -0.48 to -0.01) and switching to 5 or more days a week of physical activity a -1.04% lower body fat (CI -1.25 to -0.82). Switching from a private mode to an active mode of travel was associated with a -0.55% lower percentage body fat (CI -0.80 to -0.31) and switching to public transport with a -0.43% lower percentage body fat CI (-0.75 to -0.12).

Associations of changes in travel mode with BMI across our markers of SEP are shown in **Figure 1** and **Appendix Table 3.** Interaction tests did not reveal differences between income groups (p=0.130) nor NS-SEC (p=0.074). However, visual interpretation of these charts did appear to show that these relationships were most concentrated in the lower SEP groups. For example, switching to active travel was associated with a -0.32kg/m^2 lower BMI (CI -0.58 to -0.06) among those in the lowest household income group compared with -0.11kg/m^2 among the highest household income group (CI -0.24 to 0.03). Similarly switching to active travel was associated with a -0.33kg/m^2 lower BMI (CI -0.55 to -0.11) among those in the *Not Economically Active* NS-SEC group, but a -0.09kg/m^2 lower BMI (CI -0.22, 0.04) among those in *Managerial/Professional* NS-SEC group.

Results were similar for percentage body fat and are shown in **Figure 2** and **Appendix Table 4**. Interaction tests did not reveal there to be statistically significant differences in associations between income groups (p=0.908) nor NS-SEC groups (p=0.128). Switching to active travel was associated with a -0.71% lower body fat (-1.47, 0.05) among children in the lowest household income group, compared with a -0.55% lower body fat (-1.01, -0.09) among those in the highest income group. Among the *Economically Inactive* NS-SEC group, switching to an active travel mode was associated with a -0.78% lower body fat (-1.27, -0.29) compared with a -0.43% lower body fat (-0.78, -0.08) among the M*anagerial/Professional* NS-SEC group.

Exploratory analyses of the potential role of journey duration to school in findings by SEP were performed for individuals walking or cycling to school. These found that more affluent children were more likely to travel more than 15 minutes each way to school. 60.5% of children in the M*anagerial/Professional* NS-SEC group travelled 15 minutes or further compared with 51.9% of those in the N*ot Economically Active* NS-SEC group (Adjusted Odds Ratio (AOR) 1.18, CI 1.04 to 1.34). 60.7% of children in the highest income households travelled 15 minutes or more to school compared with 48.6% of those in the lowest income households (AOR 1.21, 95%CI 1.03 to 1.42). Analyses interacting duration with mode of travel were restricted to only ages 11 and age 14 due to data availability, but interaction tests did not reveal a difference between the associations for

For peer review only

walkers/cyclists more than vs. less than 15 minutes away for BMI (p= 0.259) nor body fat (p=0.452) (**Appendix Table 5**).

Discussion

This longitudinal study followed children over a key period in the lifecourse, and found that switching from private motorised modes of travel to school to active travel was associated with reduced objectively-measured adiposity. Additionally, switching to the use of public transport was associated with lower adiposity, although associations with BMI did not reach statistical significance. There was also a suggestion that these associations may be more concentrated among the most disadvantaged children, who are at greater risk of excess adiposity and relevant sequelae.

Strengths and limitations

This study used an established and representative UK birth cohort study with high quality data following up the same children over 8 years. We used two objective measures of adiposity and employed models that produce robust estimates of within-individual changes, which improves confidence in these results. We were able to adjust for unobserved time-invariant individual characteristics that can bias the relationship between travel mode and adiposity. Nonetheless, there are limitations to this research which should be considered. We rely on self-reported mode of travel to school, which while it was consistent across the study period, did not capture information on multi-mode journeys or variation in different modes across the week. This may have introduced some misclassification in our findings. Our reliance on these self-report questions is in contrast to some other research using the Millennium Cohort Study, which has used objective physical activity measures by accelerometers [25]. However, issues with using accelerometer data here include that is currently not available for the age 14 sweep; sample sizes being substantially lower for this method of data collection; and difficulties in ascertaining cycling to school. There was an average of three years between data collection points, and we do not know at what point between data collections individuals changed their travel behaviour. While we were able to consider a wide range of covariates, there may be some residual confounding from time-varying factors not considered [26]. We have included some exploratory analyses of whether there are inequalities in the time taken to get to school. For these, the distance between home and school may have served as a more accurate marker of physical activity dose, although this data was not available. There is also the potential for some confounding from other lifestyle factors as, for example, individuals who switch

For peer review only

to more active modes may be choosing to lead a healthier lifestyle in other ways also, and changes in adiposity may be occurring via alternative mechanisms than active travel.

Comparison with other research and practical implications

This study contributes to the evidence base by using a robust longitudinal examination of changes in travel modes and adiposity as well as specifically investigating whether there are differences across markers of SEP. We use a national sample and we were able to employ more robust methods, control for more potential confounding factors and follow children up for longer compared to many previous studies. The primary finding of this study is that switching to using an active mode of travel was linked to lower adiposity which add to the evidence base that active forms of travel to school are linked with more favourable levels of adiposity [11][27][28]. A review in 2011 found a limited number of studies but concluded that active travel to school was associated with greater cardiorespiratory fitness and that increased physical activity is a likely pathway [11].The second main finding of this study is that switching to public transport was linked to reduced adiposity although associations with BMI did not reach statistical significance. There has been limited consideration of the impacts of public transportation use on health, but this finding does broadly concur with a recent systematic review in adults which identified 10 relevant studies. This systematic review found that switching to public transport was associated with a -0.30 decrease in BMI, which is larger than our point estimate of -0.13, albeit in adults [29]. We also find no statistical evidence of differences between SEP groups, although there was a trend towards larger effects in more deprived groups. However, we caution against over interpretation of this finding given our restriction to only examining within-individual variation (hence reducing our statistical power) and the limited number of individuals within the study after stratifying by markers of SEP.

Nonetheless, active travel is only one potential mechanism to reduce adiposity in the population, and our findings also point to beneficial impacts of eating breakfast every day and sporting physical activity. For example, the associations identified here for switching to active forms of travel were considerably smaller than for switching to 5 or more days per week of physical activity for both BMI (-0.22kg/m^2 vs. -0.32kg/m^2) and body fat (-0.55% vs. -1.00%). Similarly, switching to being physically active ≥5 days per week was associated with larger changes in adiposity (e.g. 1.04% of body fat). Both of these findings concur with a wealth of other evidence on the importance of physical activity and dietary behaviours, both using MCS data and [16][30].

While we find higher levels of active travel use among more deprived groups, the wider evidence base on physical activity by markers of deprivation presents a mixed picture. Research using

For peer review only

objective physical activity data from the MCS found that when they were aged seven, children in more deprived areas were more likely to be obese, but were also more likely to hit recommended levels of physical activity [31]. Recent reviews have questioned whether there are differences in physical activity by deprivation, and concluded that while more affluent children do more leisure time activity, overall differences remain small for physical activity overall and for active travel specifically [32].

These findings highlight the potential for a shift to physically active modes of travel to play a role in reducing population level adiposity, and potentially to reduce health inequalities. They also reinforce calls from the World Health Organisation to develop policies which promote incorporating physical activity into daily lives [33]. However, there is mixed evidence on the how best to increase levels of active travel to school, which remains a fruitful avenue for future research [34]. Additionally, determinants of active travel are known to be wide ranging including parental concerns regarding safety, attitudes of the school and wider environmental factors [35][36][37][38][39]. Systematic reviews have found a wide range of potential determinants, including negative associations with traffic volumes, but that results were heterogeneous due to differing contexts and measures [40]. This means that there is a need for context-specific research alongside practical actions to increase the use of physically active travel modes. These will likely need to act at a number of levels, including addressing availability of safe cycle routes, school-level policies to discourage driving and national policies to improve infrastructure. Given the well-established benefits of physical activity on outcomes other than adiposity, and the potential additional benefits of increasing active travel in the form of air pollution and greenhouse gases, concerted action should be made to encourage the use of more active modes of travel to school [41].

For peer review only

Conclusion

This seven-year longitudinal study with data collected at three time points has concluded that switching to active forms of travel can have beneficial adiposity impacts at a key time in the lifecourse. These benefits may be more apparent among disadvantaged children. This research adds to the evidence base on the health impacts of active travel and suggests that targeted structural interventions may reduce inequalities in health in the UK.

Declarations

Patient and Public Involvement

Patients were not involved in the design of this study

Ethical approval

As this study involved secondary analysis the MCS ethical approval was not required. Data collection for the MCS has ethical approval from the Yorkshire and Humber ethics committee (11/YH/0203) and further details are available from http://www.cls.ioe.ac.uk/.

Consent for publication

Not applicable

Availability of data and materials

The datasets generated during and/or analysed during the current study are available in the UKDataService repository https://ukdataservice.ac.uk/

Competing interests

All authors declare that they have no competing interests

Funding

This study/project is funded by the National Institute for Health Research (NIHR) School for Public Health Research (Grant Reference Number PD-SPH-2015) as well as a NIHR Research Professorship award to Professor Millett (NIHR RP_2014-04-032). The views expressed are those of the author(s) and not necessarily those of the NIHR or the Department of Health and Social Care

Authors' contributions

AL and CM conceived of the study. AL, CM and TH devised the analyses. AL conducted the analyses with the assistance of TH. AL produced the initial draft and TH, CM, AG and YK revised this for significant intellectual content and approve the final version

Acknowledgements

We would like to acknowledge the participants of the MCS

For peer review only

Table 1: Mode of travel to school in the Millennium Cohort Study in 2014/15, age 14/15 years

		Number	Car % (number)	PT % (number)	Active % (number)
Gender	Male	4,102	24.2 (992)	35.4 (1453)	40.4 (1657)
	Female	4,330	27.9 (1206)	35.2 (1526)	36.9 (1598)
Country	England	5,409	26.9 (1455)	29.8 (1612)	43.3 (2342)
	Wales	1,182	25.5 (302)	40.3 (476)	34.2 (404)
	Scotland	958	20.0 (192)	40.5 (388)	39.5 (378)
	Northern Ireland	883	28.2 (249)	57.0 (503)	14.8 (131)
NS-SEC social class	Not economically active	2,653	27.5 (531)	37.2 (657)	35.2 (827)
	Lower	2,054	26.5 (392)	38.3 (547)	35.2 (771)
	Intermediate	1,710	22.9 (545)	32.0 (787)	45.1 (722)
	Managerial/professional	2,015	26.4 (730)	32.6 (988)	41.0 (935)
Household income group	Lowest	1,092	26.1 (285)	27.9 (305)	46 (502)
	Second lowest	1,286	23.2 (298)	36.5 (469)	40.4 (519)
	Middle	1,737	25.2 (438)	37.2 (646)	37.6 (653)
	Second highest	2,121	27.3 (578)	35.7 (757)	37.1 (786)
	Highest	2,196	27.3 (599)	36.5 (802)	36.2 (795)
Ethnic group	White	7,329	25 (1832)	36.5 (2677)	38.5 (2820)
	Mixed	64	17.2 (11)	45.3 (29)	37.5 (24)
	Indian	208	34.6 (72)	26.4 (55)	38.9 (81)
	Pakistani or Bangladeshi	468	42.7 (200)	10.9 (51)	46.4 (217)
	Black or Black British	241	18.7 (45)	49.4 (119)	32.0 (77)
	Other	122	31.1 (38)	39.3 (48)	29.5 (36)
Growth spurt	Not yet/barely started	2,952	25.4 (750)	35.9 (1060)	38.7 (1142)
	Begun or completed	5,480	26.4 (1448)	35.0 (1919)	38.6 (2113)
Breakfast consumption	< 7 days per week	3,824	25.8 (985)	35.0 (1339)	39.2 (1500)
	Every day	4,608	26.3 (1213)	35.6 (1640)	38.1 (1755)
Portions of fruit per day	≤3 portions per day	5,775	26.1 (1510)	34.5 (1994)	39.3 (2271)
	≥2 portions per day	2,655	25.9 (688)	37.1 (985)	37.0 (982)
Physical activity	≤2 days per week	2,342	27.8 (650)	34.3 (804)	37.9 (888)
	3-4 days per week	2,905	25.0 (725)	37.6 (1092)	37.5 (1088)
	≥ 5 days per week	3,185	25.8 (823)	34.0 (1083)	40.2 (1279)
TV or computer use	≤2 hours per day	1,897	26.4 (501)	38.2 (724)	35.4 (672)
	≥3 hours per day	6,535	26.0 (1697)	34.5 (2255)	39.5 (2583)
Overall		8,432	26.1 (2198)	35.3 (2979)	38.6 (3255)

PT = Public transport, NS-SEC = National Statistics Socio-Economic Classification

For peer review only

Table 2: Results from longitudinal fixed-effects regression of impacts of switching school travel mode and adiposity

		BMI		% Body fat	
		coeff	95% CI	coeff	95% CI
Mode of travel	Private transport	ref	ref	ref	ref
	Public transport	-0.13	-0.26 ;0.01	-0.43	-0.75 ;-0.12
	Walk/Cycle	-0.21	-0.31 ;-0.10	-0.55	-0.80 ;-0.31
Age	7	ref	ref	ref	ref
	11	2.44	2.37 ;2.52	1.06	0.87 ;1.25
	14	4.59	4.45 ;4.73	0.21	-0.14 ;0.56
Country	England	ref	ref	ref	ref
	Wales	0.13	-1.10 ;1.37	1.77	-0.54 ;4.08
	Scotland	0.01	-1.00 ;1.01	0.46	-1.88 ;2.79
	Northern Ireland	-0.10	-1.92 ;1.72	1.07	-3.04 ;5.19
NS-SEC social class	Not economically active	ref	ref	ref	ref
	Lower	-0.04	-0.18 ; 0.10	-0.22	-0.53 ; 0.10
	Intermediate	-0.08	-0.21 ; 0.06	-0.24	-0.58 ; 0.07
	Managerial/professional	0.08	-0.06 ; 0.22	0.16	-0.18 ; 0.50
Household income group	Lowest	ref	ref	ref	ref
	Second lowest	0.08	-0.07 ;0.23	0.41	0.05 ;0.77
	Middle	0.13	-0.03 ;0.29	0.36	-0.02 ;0.75
	Second highest	0.06	-0.11 ;0.23	0.12	-0.30 ;0.54
	Highest	-0.01	-0.20 ;0.17	0.14	-0.30 ;0.59
Growth spurt	Not yet/barely started	ref	ref	ref	ref
	Begun or completed	0.22	0.14 ;0.31	0.1	-0.11 ;0.31
Breakfast consumption	< 7 days per week	ref	ref	ref	ref
	Every day	-0.39	-0.51 ;-0.27	-1.34	-1.62 ;-1.06
Portions of fruit per day	≤3 portions per day	ref	ref	ref	ref
	≥2 portions per day	0.05	-0.04 ;0.15	0.11	-0.11 ;0.33
Physical activity	≤2 days per week	ref	ref	ref	ref
	3-4 days per week	-0.06	-0.16 ;0.04	-0.25	-0.48 ;-0.01
	≥ 5 days per week	-0.32	-0.41 ;-0.23	-1.04	-1.25 ;-0.82
TV or computer use	≤2 hours per day	ref	ref	ref	ref
	≥3 hours per day	0.07	-0.02 ;0.16	0.22	-0.01 ;0.44

Results from one separate model for BMI and one separate model for percentage body fat. Models adjusted for individual-level fixed effects and changes in: country; highest NSSEC in household; household income; eating breakfast; frequency of eating breakfast; self-reported growth spurt; hours of TV and computer use per day; and days per week of moderate-to-vigorous physical activity. Coeff = coefficient, ref = reference category

For peer review only

Figure 1: Changes in BMI and 95% Confidence Intervals by mode of travel to school

Caption

Results from longitudinal fixed-effects regression of impacts of switching school travel mode and BMI stratified by markers of Socio-Economic Position. These represent the average effect of switching to using active transport from any other mode

Figure 2: Changes in % body fat and 95% Confidence Intervals by mode of travel to school

Caption

Results from longitudinal fixed-effects regression of impacts of switching school travel mode and percentage body fat stratified by markers of Socio-Economic Position. These represent the average effect of switching to using active transport from any other mode

For peer review only

References

1 Ekelund U, Brage S, Froberg K, *et al.* TV viewing and physical activity are independently associated with metabolic risk in children: The European youth heart study. *PLoS Med* Published Online First: 2006. doi:10.1371/journal.pmed.0030488

2 Griffiths LJ, Cortina-Borja M, Sera F, *et al.* How active are our children? Findings from the millennium cohort study. *BMJ Open* Published Online First: 2013. doi:10.1136/bmjopen-2013-002893

3 Guthold R, Stevens GA, Riley LM, *et al.* Global trends in insufficient physical activity among adolescents: a pooled analysis of 298 population-based surveys with 1·6 million participants. *Lancet Child Adolesc Heal* Published Online First: 2020. doi:10.1016/S2352-4642(19)30323-2

4 Kwon S, Janz KF, Cooper A, *et al.* Tracking of accelerometry-measured physical activity during childhood: ICAD pooled analysis. *Int J Behav Nutr Phys Act* Published Online First: 2012. doi:10.1186/1479-5868-9-68

5 Hills AP, Andersen LB, Byrne NM. Physical activity and obesity in children. *Br J Sports Med* 2011;**45**:866 LP – 870. doi:10.1136/bjsports-2011-090199

6 Cooper AR, Goodman A, Page AS, *et al.* Objectively measured physical activity and sedentary time in youth: The International children's accelerometry database (ICAD). *Int J Behav Nutr Phys Act* Published Online First: 2015. doi:10.1186/s12966-015-0274-5

7 Morton KL, Corder K, Suhrcke M, *et al.* School polices, programmes and facilities, and objectively measured sedentary time, LPA and MVPA: Associations in secondary school and over the transition from primary to secondary school. *Int J Behav Nutr Phys Act* Published Online First: 2016. doi:10.1186/s12966-016-0378-6

8 Flint E, Cummins S, Sacker A. Associations between active commuting, body fat, and body mass index: population based, cross sectional study in the United Kingdom. *Br Med J* 2014;**349**. doi:10.1136/bmj.g4887

9 Roth MA, Millett CJ, Mindell JS. The contribution of active travel (walking and cycling) in children to overall physical activity levels: A national cross sectional study. *Prev Med (Baltim)* Published Online First: 2012. doi:10.1016/j.ypmed.2011.12.004

10 Colley R, Larouche R, Saunders TJ, *et al.* Associations between Active School Transport and Physical Activity, Body Composition, and Cardiovascular Fitness: A Systematic Review of 68 Studies. *J Phys Act Heal* Published Online First: 2016. doi:10.1123/jpah.2011-0345

11 Lubans DR, Boreham CA, Kelly P, *et al.* The relationship between active travel to school and health-related fitness in children and adolescents: A systematic review. Int. J. Behav. Nutr. Phys. Act. 2011. doi:10.1186/1479-5868-8-5

12 Department for Transport (2015). National Travel Survey 2014: Travel to school https://assets.publishing.service.gov.uk/government/uploads/system/uploads/attachment_data/file/476635/travel-to-school.pdf.

13 Pouliou T, Sera F, Griffiths L, *et al.* Environmental influences on children's physical activity. *J Epidemiol Community Health* Published Online First: 2015. doi:10.1136/jech-2014-204287

14 Laverty AA, Mindell JS, Webb EA, *et al.* Active travel to work and cardiovascular risk factors in the united kingdom. *Am J Prev Med* 2013;**45**:282–8. doi:10.1016/j.amepre.2013.04.012

15 Higgins, V., Dale, A., 2010. Ethnic differences in physical activity and obesity, working paper. Cathie Marsh Centre for Census and Survey Research https://lemosandcrane.co.uk/resources/University%20of%20Manchester%20-%20Ethnic%20differences%20in%20physic.

16 Goisis A, Sacker A, Kelly Y. Why are poorer children at higher risk of obesity and overweight? A UK cohort study. *Eur J Public Health* Published Online First: 2016. doi:10.1093/eurpub/ckv219

17 Cavill N, Davis A, Cope A, Corner D, 2019, Active Travel and Physical Activity Evidence Review. Sport England https://www.sportengland.org/media/13943/active-travel-full-report-evidence-review.pdf.

For peer review only

18 Su JG, Jerrett M, McConnell R, *et al.* Factors influencing whether children walk to school. *Health Place* 2013;**22**:153–61. doi:https://doi.org/10.1016/j.healthplace.2013.03.011

19 Noonan RJ, Boddy LM, Knowles ZR, *et al.* Fitness, fatness and active school commuting among liverpool schoolchildren. *Int J Environ Res Public Health* Published Online First: 2017. doi:10.3390/ijerph14090995

20 Schoeppe S, Duncan MJ, Badland H, *et al.* Associations of children's independent mobility and active travel with physical activity, sedentary behaviour and weight status: A systematic review. *J Sci Med Sport* 2013;**16**:312–9. doi:https://doi.org/10.1016/j.jsams.2012.11.001

21 Saunders LE, Green JM, Petticrew MP, *et al.* What Are the Health Benefits of Active Travel? A Systematic Review of Trials and Cohort Studies. *PLoS One* 2013;**8**:e69912. doi:10.1371/journal.pone.0069912

22 Connelly R, Platt L. Cohort profile: UK Millennium Cohort Study (mcs). *Int J Epidemiol* 2014;**43**:1719–25. doi:10.1093/ije/dyu001

23 Wooldridge JM (2010). Econometric analysis of cross section and panel data: MIT press; 2010.

24 Rabe-Hesketh S, Skrondal A (2008). Multilevel and longitudinal modeling using Stata: STATA press; 2008.

25 Griffiths LJ, Sera F, Cortina-Borja M, *et al.* Objectively measured physical activity and sedentary time: cross-sectional and prospective associations with adiposity in the Millennium Cohort Study. *BMJ Open* 2016;**6**:e010366. doi:10.1136/bmjopen-2015-010366

26 Harrison F, Goodman A, van Sluijs EMF, *et al.* Weather and children's physical activity; how and why do relationships vary between countries? *Int J Behav Nutr Phys Act* 2017;**14**:74. doi:10.1186/s12966-017-0526-7

27 Schoeppe S, Duncan MJ, Badland H, *et al.* Associations of children's independent mobility and active travel with physical activity, sedentary behaviour and weight status: A systematic review. *J Sci Med Sport* 2013;**16**:312–9. doi:https://doi.org/10.1016/j.jsams.2012.11.001

28 Faulkner GEJ, Buliung RN, Flora PK, *et al.* Active school transport, physical activity levels and body weight of children and youth: A systematic review. *Prev Med (Baltim)* 2009;**48**:3–8. doi:http://dx.doi.org/10.1016/j.ypmed.2008.10.017

29 Patterson R, Webb E, Hone T, *et al.* Associations of Public Transportation Use With Cardiometabolic Health: A Systematic Review and Meta-Analysis. *Am J Epidemiol* 2019;**188**:785–95. doi:10.1093/aje/kwz012

30 World Health Organization (2020) Report of the commission on ending childhood obesity. https://apps.who.int/iris/bitstream/handle/10665/204176/9789241510066_eng.pdf;jsessionid=F36FB7899AA415F5EDC15F7D35B4B230?sequence=1.

31 Noonan RJ, Fairclough SJ. Is there a deprivation and maternal education gradient to child obesity and moderate-to-vigorous physical activity? Findings from the Millennium Cohort Study. *Pediatr Obes* 2018;**13**:458–64. doi:10.1111/ijpo.12287

32 Stalsberg R, Pedersen AV. Are Differences in Physical Activity across Socioeconomic Groups Associated with Choice of Physical Activity Variables to Report? *Int J Environ Res Public Health* 2018;**15**:922. doi:10.3390/ijerph15050922

33 World Health Organization. Global action plan for the prevention and control of NCDs 2013-2020. Published Online First: 2013.http://www.who.int/nmh/publications/ncd-action-plan/en/

34 Larouche R, Mammen G, Rowe DA, *et al.* Effectiveness of active school transport interventions: a systematic review and update. *BMC Public Health* 2018;**18**:206. doi:10.1186/s12889-017-5005-1

35 Garnham-Lee KP, Falconer CL, Sherar LB, *et al.* Evidence of moderation effects in predicting active transport to school. *J Public Health (Oxf)* 2017;**39**:153–62. doi:10.1093/pubmed/fdw016

36 Panter J, Corder K, Griffin SJ, *et al.* Individual, socio-cultural and environmental predictors of uptake and maintenance of active commuting in children: longitudinal results from the

For peer review only

SPEEDY study. *Int J Behav Nutr Phys Act* 2013;**10**:83. doi:10.1186/1479-5868-10-83

37 Carver A, Salmon J, Campbell K, *et al.* How Do Perceptions of Local Neighborhood Relate to Adolescents' Walking and Cycling? *Am J Heal Promot* 2005;**20**:139–47. doi:10.4278/0890-1171-20.2.139

38 Panter JR, Jones AP, van Sluijs EMF, *et al.* Attitudes, social support and environmental perceptions as predictors of active commuting behaviour in school children. *J Epidemiol Community Health* 2010;**64**:41–8. doi:10.1136/jech.2009.086918

39 Morton KL, Atkin AJ, Corder K, *et al.* The school environment and adolescent physical activity and sedentary behaviour: a mixed-studies systematic review. *Obes Rev* 2016;**17**:142–58. doi:10.1111/obr.12352

40 Sharmin S, Kamruzzaman M. Association between the built environment and children's independent mobility: A meta-analytic review. *J Transp Geogr* 2017;**61**:104–17. doi:https://doi.org/10.1016/j.jtrangeo.2017.04.004

41 Woodcock J, Edwards P, Tonne C, *et al.* Public health benefits of strategies to reduce greenhouse-gas emissions: urban land transport. *Lancet* 2009;**374**:1930–43. doi:S0140-6736(09)61714-1 [pii] 10.1016/S0140-6736(09)61714-1

For peer review only

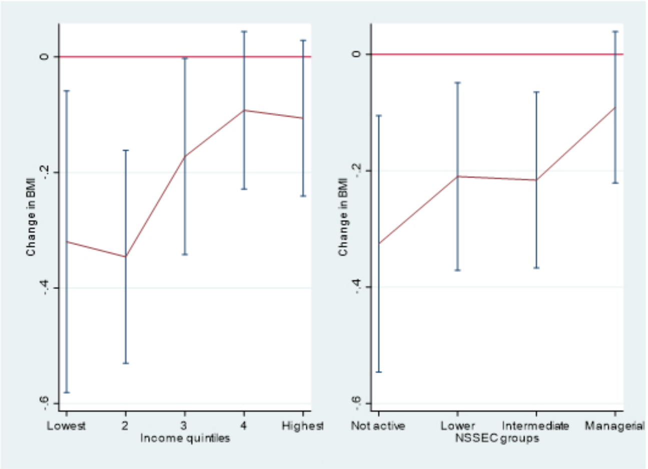

Figure 1: Changes in BMI and 95% Confidence Intervals by mode of travel to school

216x121mm (96 x 96 DPI)

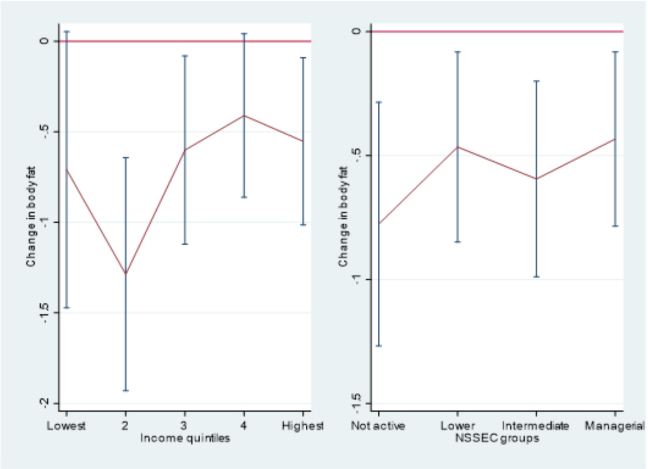

Figure 2: Changes in % body fat and 95% Confidence Intervals by mode of travel to school

216x121mm (96 x 96 DPI)

Appendix table 1: Mode of travel to school in the Millennium Cohort Study in 2011/12, age 10-11 years

		Car	PT	Active
Gender	Male	47.4	5.3	47.3
	Female	47.2	5.2	47.6
Country	England	45.5	2.6	51.9
	Wales	51.1	7.6	41.3
	Scotland	37.8	9.8	52.4
	Northern Ireland	62.9	13.9	23.1
NS-SEC social class	Not economically active	28.7	4.9	66.4
	Lower	39.7	5.7	54.6
	Intermediate	53.7	4.9	41.4
	Managerial/professional	53.7	5.1	41.2
Household income group	Lowest	34.6	5.5	59.9
	Second lowest	43.3	6.6	50.0
	Middle	47.8	5.2	47.0
	Second highest	51.1	4.5	44.4
	Highest	56.2	4.7	39.1
Ethnic group	White	48.0	5.7	46.3
	Mixed	41.6	4.7	53.7
	Indian	58.4	1.0	40.6
	Pakistani or Bangladeshi	38.1	1.1	60.8
	Black or Black British	40.5	6.2	53.3
	Other	43.5	3.3	53.3
Growth spurt*	Not yet/barely started	-	-	-
	Begun or completed	-	-	-
Breakfast consumption	< 7 days per week	42.9	7.3	49.8
	Every day	47.6	5.1	47.3
Portions of fruit per day	≤3 portions per day	46.1	5.1	48.8
	≥2 portions per day	47.6	5.3	47.1
Physical activity	≤2 days per week	48.9	4.2	46.9
	3-4 days per week	50.1	5.0	44.9
	≥ 5 days per week	46.2	5.7	48.1
TV or computer use	≤2 hours per day	47.5	5.5	47.0
	≥3 hours per day	46.4	4.2	49.4
Overall		47.3	5.3	47.4

PT = Public Transport , * N/A

For peer review only

Appendix table 2: Mode of travel to school in the Millennium Cohort Study in 2007/08, age 7-8 years

		Car	PT	Active
Gender	Male	42.6	6.6	50.9
	Female	44.7	6.9	48.4
Country	England	42.1	4.7	53.2
	Wales	47.1	8.4	44.5
	Scotland	32.2	10.1	57.8
	Northern Ireland	60.8	13.6	25.6
NS-SEC social class	Not economically active	36.8	6.8	56.4
	Lower	38.3	5.9	55.7
	Intermediate	47.8	6.4	45.8
	Managerial/professional	50.7	7.4	41.8
Household income group	Lowest	31.0	6.4	62.6
	Second lowest	40.2	8.2	51.7
	Middle	48.5	6.8	44.7
	Second highest	48.7	6.0	45.3
	Highest	46.3	6.4	47.3
Ethnic group	White	43.9	6.9	49.2
	Mixed	42.2	10.1	47.7
	Indian	55.3	2.6	42.1
	Pakistani or Bangladeshi	41.3	1.8	56.9
	Black or Black British	33.6	12.3	54.0
	Other	40.0	5.6	54.4
Growth spurt	Not yet/barely started	45.0	6.5	48.5
	Begun or completed	42.3	7.0	50.7
Breakfast consumption	< 7 days per week	40.0	8.5	51.5
	Every day	44.2	6.5	49.3
Portions of fruit per day	≤3 portions per day	42.3	7.3	50.5
	≥2 portions per day	44.3	6.5	49.2
Physical activity	≤2 days per week	44.7	6.8	48.5
	3-4 days per week	44.4	6.6	49.0
	≥ 5 days per week	42.7	6.7	50.6
TV or computer use	≤2 hours per day	44.5	7.1	48.4
	≥3 hours per day	39.9	5.0	55.1
Overall		43.7	6.7	49.6

For peer review only

Appendix Table 3: Results from longitudinal fixed-effects regression of impacts of switching school travel mode and BMI stratified by markers of Socio-Economic Position

NSSEC groups	Coefficient	Lower CI	Upper CI
Managerial/professional	-0.09	-0.22	0.04
Self-employed intermediate	-0.22	-0.37	-0.07
Lower semi-routine routine	-0.21	-0.37	-0.05
Not economically active	-0.33	-0.55	-0.11
Household income groups			
Highest quantile income	-0.11	-0.24	0.03
Fourth quantile	-0.09	-0.23	0.04
Third quantile	-0.17	-0.34	0.00
Second quantile	-0.35	-0.53	-0.16
Lower quantile income	-0.32	-0.58	-0.06

NSSEC = National Statistics Socio-economic Classification, CI = Confidence Interval

Models adjusted for individual-level fixed effects and changes in: country; highest NSSEC in household; household income; eating breakfast; frequency of eating breakfast; self-reported growth spurt; hours of TV and computer use per day; and days per week of moderate-to-vigorous physical activity.

Appendix Table 4: Results from longitudinal fixed-effects regression of impacts of switching school travel mode and percentage body fat stratified by markers of Socio-Economic Position

NSSEC groups	Coefficient	Lower CI	Upper CI
Managerial/professional	-0.43	-0.78	-0.08
Self-employed intermediate	-0.59	-0.99	-0.20
Lower semi-routine routine	-0.47	-0.85	-0.08
Not economically active	-0.78	-1.27	-0.29
Household income groups			
Highest quantile income	-0.55	-1.01	-0.09
Fourth quantile	-0.41	-0.86	0.04
Third quantile	-0.60	-1.12	-0.08
Second quantile	-1.29	-1.93	-0.64
Lower quantile income	-0.71	-1.47	0.05

Models adjusted for individual-level fixed effects and changes in: country; highest NSSEC in household; household income; eating breakfast; frequency of eating breakfast; self-reported growth spurt; hours of TV and computer use per day; and days per week of moderate-to-vigorous physical activity.

For peer review only

Appendix table 5: Fully adjusted odds ratios travelling 15 minutes or more each way by walking or cycling

		%	OR	Lower CI	Upper CI
Gender	Male	42.2	ref	ref	ref
	Female	45.1	1.14	1.00	1.30
Country	England	45.2	ref	ref	ref
	Wales	38.5	0.65	0.53	0.79
	Scotland	41.2	0.74	0.61	0.91
	Northern Ireland	36.6	0.60	0.43	0.83
NS-SEC social class	Not economically active	39.7	ref	ref	ref
	Lower	43.8	1.10	0.91	1.33
	Intermediate	44.5	1.07	0.87	1.32
	Managerial/professional	47.0	1.20	0.98	1.47
Household income group	Lowest	38.2	ref	ref	ref
	Second lowest	43.8	1.00	0.80	1.25
	Middle	44.9	0.98	0.77	1.23
	Second highest	44.9	0.88	0.69	1.11
	Highest	46.0	0.86	0.66	1.11
Ethnic group	White	46.2	ref	ref	ref
	Mixed	53.8	1.30	0.67	2.53
	Indian	36.4	0.60	0.41	0.88
	Pakistani or Bangladeshi	23.9	0.30	0.23	0.40
	Black or Black British	38.9	0.66	0.45	0.97
	Other	32.3	0.53	0.31	0.90
Growth spurt	Not yet/barely started	43.3	ref	ref	ref
	Begun or completed	44.2	0.99	0.87	1.13
Breakfast consumption	< 7 days per week	43.3	ref	ref	ref
	Every day	44.0	1.07	0.94	1.22
Portions of fruit per day	≤3 portions per day	43.4	ref	ref	ref
	≥2 portions per day	44.4	1.00	0.87	1.16
Physical activity	≤2 days per week	42.7	ref	ref	ref
	3-4 days per week	45.6	1.14	0.97	1.34
	≥ 5 days per week	42.5	0.98	0.83	1.15
TV or computer use	≤2 hours per day	42.5	ref	ref	ref
	≥3 hours per day	43.9	1.01	0.86	1.18

For peer review only

STROBE 2007 (v4) Statement—Checklist of items that should be included in reports of *cohort studies*

Section/Topic	Item #	Recommendation	Reported on page #
**Title and abstract**	1	(*a*) Indicate the study's design with a commonly used term in the title or the abstract	1
		(*b*) Provide in the abstract an informative and balanced summary of what was done and what was found	1 - 2
**Introduction**			
Background/rationale	2	Explain the scientific background and rationale for the investigation being reported	4 - 5
Objectives	3	State specific objectives, including any prespecified hypotheses	5
**Methods**			
Study design	4	Present key elements of study design early in the paper	4 - 5
Setting	5	Describe the setting, locations, and relevant dates, including periods of recruitment, exposure, follow-up, and data collection	5 - 6
Participants	6	(*a*) Give the eligibility criteria, and the sources and methods of selection of participants. Describe methods of follow-up	5 - 6
		(*b*) For matched studies, give matching criteria and number of exposed and unexposed	n / a
Variables	7	Clearly define all outcomes, exposures, predictors, potential confounders, and effect modifiers. Give diagnostic criteria, if applicable	5 - 7
Data sources/ measurement	8*	For each variable of interest, give sources of data and details of methods of assessment (measurement). Describe comparability of assessment methods if there is more than one group	*6*
Bias	9	Describe any efforts to address potential sources of bias	6
Study size	10	Explain how the study size was arrived at	5
Quantitative variables	11	Explain how quantitative variables were handled in the analyses. If applicable, describe which groupings were chosen and why	5 - 6
Statistical methods	12	(*a*) Describe all statistical methods, including those used to control for confounding	5 – 6
		(*b*) Describe any methods used to examine subgroups and interactions	6
		(*c*) Explain how missing data were addressed	6
		(*d*) If applicable, explain how loss to follow-up was addressed	6
		(*e*) Describe any sensitivity analyses	6
**Results**			

For peer review only

Participants	13*	(a) Report numbers of individuals at each stage of study—eg numbers potentially eligible, examined for eligibility, confirmed eligible, included in the study, completing follow-up, and analysed	6
		(b) Give reasons for non-participation at each stage	5 – 6
		(c) Consider use of a flow diagram	n / a
Descriptive data	14*	(a) Give characteristics of study participants (eg demographic, clinical, social) and information on exposures and potential confounders	Table 1
		(b) Indicate number of participants with missing data for each variable of interest	Table 1
		(c) Summarise follow-up time (eg, average and total amount)	5 - 6
Outcome data	15*	Report numbers of outcome events or summary measures over time	7
Main results	16	(*a*) Give unadjusted estimates and, if applicable, confounder-adjusted estimates and their precision (eg, 95% confidence interval). Make clear which confounders were adjusted for and why they were included	7
		(*b*) Report category boundaries when continuous variables were categorized	7 – 9
		(*c*) If relevant, consider translating estimates of relative risk into absolute risk for a meaningful time period	8
Other analyses	17	Report other analyses done—eg analyses of subgroups and interactions, and sensitivity analyses	8
**Discussion**			
Key results	18	Summarise key results with reference to study objectives	9
**Limitations**			
Interpretation	20	Give a cautious overall interpretation of results considering objectives, limitations, multiplicity of analyses, results from similar studies, and other relevant evidence	9
Generalisability	21	Discuss the generalisability (external validity) of the study results	10
**Other information**			
Funding	22	Give the source of funding and the role of the funders for the present study and, if applicable, for the original study on which the present article is based	12

*Give information separately for cases and controls in case-control studies and, if applicable, for exposed and unexposed groups in cohort and cross-sectional studies.

Note: An Explanation and Elaboration article discusses each checklist item and gives methodological background and published examples of transparent reporting. The STROBE checklist is best used in conjunction with this article (freely available on the Web sites of PLoS Medicine at http://www.plosmedicine.org/, Annals of Internal Medicine at http://www.annals.org/, and Epidemiology at http://www.epidem.com/). Information on the STROBE Initiative is available at www.strobe-statement.org.

For peer review only

Associations of active travel with adiposity among children and socio-economic differentials: a longitudinal study

Journal:	*BMJ Open*
Manuscript ID	bmjopen-2019-036041.R2
Article Type:	Original research
Date Submitted by the Author:	16-Nov-2020
Complete List of Authors:	Laverty, Anthony; Imperial College London<br>Hone, Thomas; Imperial College London,<br>Goodman, Anna; LSHTM, Faculty of Epidemiology and Population Health<br>Kelly, Yvonne; University College London, Epidemiology and Public Health<br>Millett, Christopher; Imperial College London
**Primary Subject Heading**:	Public health
Secondary Subject Heading:	Health policy
Keywords:	PUBLIC HEALTH, Community child health < PAEDIATRICS, PREVENTIVE MEDICINE

For peer review only - http://bmjopen.bmj.com/site/about/guidelines.xhtml

BMJ

I, the Submitting Author has the right to grant and does grant on behalf of all authors of the Work (as defined in the below author licence), an exclusive licence and/or a non-exclusive licence for contributions from authors who are: i) UK Crown employees; ii) where BMJ has agreed a CC-BY licence shall apply, and/or iii) in accordance with the terms applicable for US Federal Government officers or employees acting as part of their official duties; on a worldwide, perpetual, irrevocable, royalty-free basis to BMJ Publishing Group Ltd ("BMJ") its licensees and where the relevant Journal is co-owned by BMJ to the co-owners of the Journal, to publish the Work in this journal and any other BMJ products and to exploit all rights, as set out in our licence.

The Submitting Author accepts and understands that any supply made under these terms is made by BMJ to the Submitting Author unless you are acting as an employee on behalf of your employer or a postgraduate student of an affiliated institution which is paying any applicable article publishing charge ("APC") for Open Access articles. Where the Submitting Author wishes to make the Work available on an Open Access basis (and intends to pay the relevant APC), the terms of reuse of such Open Access shall be governed by a Creative Commons licence – details of these licences and which Creative Commons licence will apply to this Work are set out in our licence referred to above.

Other than as permitted in any relevant BMJ Author's Self Archiving Policies, I confirm this Work has not been accepted for publication elsewhere, is not being considered for publication elsewhere and does not duplicate material already published. I confirm all authors consent to publication of this Work and authorise the granting of this licence.

For peer review only

Associations of active travel with adiposity among children and socio-economic differentials: a longitudinal study

Anthony A. Laverty[1*], Thomas Hone[1], Anna Goodman[2], Yvonne Kelly[3], Christopher Millett[1]

[1] Public Health Policy Evaluation Unit, School of Public Health, Imperial College London

[2] Faculty of Epidemiology and Population Health, London School of Hygiene and Tropical Medicine

[3] Department of Epidemiology and Public Health, 1-19 Torrington Place, University College London, London, WC1E 6BT, UK

* Corresponding author a.laverty@imperial.ac.uk

For peer review only

Objectives

Examine longitudinal associations between modes of travel to school and adiposity

Setting

UK

Participants

8,432 children surveyed at ages 7, 11 and 14 years from the UK Millennium Cohort study.

Primary and secondary outcomes

Objective percentage body fat and Body Mass Index (BMI). Transport mode was categorised as private motorised transport, public transport, active transport (walking or cycling). Socio-Economic Position (SEP) was measured by household income group and occupational social class We adjusted analyses for changes in country of United Kingdom, frequency of eating breakfast, self-reported growth spurts, *hours of screen time, and days per week of moderate-to-vigorous physical activity.* Longitudinal (panel) regression models adjusting for individual fixed effects examined associations in changes in mode of travel to school and adiposity, controlling for both time-varying and time-invariant potential confounders. Interaction tests and stratified analyses investigated differences by markers of SEP.

Results

At age 14, 26.1% of children (2,198) reported using private motorised transport, 35.3% (2979) used public transport, and 38.6% (3,255) used active transport to get to school. 36.6% (3,083) of children changed mode twice between the three waves and 50.7% (4,279) changed once. Compared with continuing to use private transport, switching to active transport was associated with a lower BMI (-0.21kg/m^2, 95% Confidence Interval -0.31;-0.10) and body fat (-0.55%, -0.80;-0.31). Switching to public transport was associated with lower percentage body fat (-0.43%, -0.75;-0.12), but associations with BMI did not reach statistical significance (-0.13kg/m2, -0.26; 0.01). Interaction tests showed a trend for these effects to be stronger in more deprived groups, but these interactions did not reach statistical significance

Conclusion

This longitudinal study during a key lifecourse period found switching to physically active forms of travel can have beneficial adiposity impacts; these associations may be more apparent for more disadvantaged children. Increasing active travel has potential to ameliorate inequalities.

For peer review only

Strengths and limitations of this study

- Used an established and representative UK birth cohort study with high quality data following up the same children over 8 years.
- We used two objective measures of adiposity and employed models that produce robust estimates of within-individual changes and were able to adjust for unobserved time-invariant individual characteristics
- However, we rely on self-reported mode of travel to school, which did not capture information on multi-mode journeys or variation in different modes across the week.
- There was an average of three years between data collection points, and we do not know at what point between data collections individuals changed their travel behaviour.
- While we were able to consider a wide range of covariates, there may be some residual confounding from time-varying factors not considered such as choosing to lead a healthier lifestyle in other ways also, and changes in adiposity may be occurring via alternative mechanisms than active travel.

For peer review only

Introduction

Insufficient physical activity is associated with increased cardio-metabolic risk, including higher levels of adiposity in children as well as adults [1]. Children are an important group for physical activity promotion and the majority of children and adolescents do not reach recommended levels [2][3]. Additionally evidence from both the UK and international studies suggests that levels of physical activity track across the lifecourse, meaning that those who are insufficiently active early in life are at risk of this continuing into later life [4][5][6]. The period of transition from primary to secondary education is characterised by significant changes to children's social and physical environment which can strongly influence health behaviours [7].

Active travel (that is, the use of non-motorised modes of travel) has the potential to increase physical activity and reduce adiposity [8]. The use of active travel in children has been linked to higher levels of physical activity in a range of settings, including during the journey to school [6][9][10][11]. Modes of travel change significantly during childhood, with the majority of travel being with caregivers in primary school and then changing to more independent modes later [12]. There are also significant differentials across socio-economic position (SEP) in physical activity profiles, use of active travel, and cardio-metabolic health risks in children, which together contribute to levels of health inequalities between groups [13][14][15]. Inequalities are also present among children for other factors and behaviours which are associated with greater risks of obesity and ill-health, including screen time and dietary behaviours [16].While active travel may have the potential to offset some of these inequalities, a recent review by Sport England concluded that there is a comparative lack of evidence on the impacts of active travel across groups across markers of SEP [17].

Much of the evidence on the impacts of active travel has been cross-sectional and there has been a lack of evidence using large-scale prospective studies, especially in children. Studies have highlighted conflicting evidence on the potential role of active travel to school on body composition and adiposity and, a recent systematic review found a limited number of prospective studies in children and did not identify an association between active travel and obesity in children [18][19][20][21]. This study adds to the evidence base by using a robust analytical strategy on data from a representative cohort of children during a key period in their development to investigate the role of active travel to school on objectively measured adiposity, and the potential for differential effects according to two different measures of SEP. Multivariate longitudinal (panel) regression models are employed to follow the same individuals over time, adjust for time invariant factors, and assess changes in transport usage and health outcomes.

For peer review only

Methods

Sample and data

Data for this study came from the Millennium Cohort Study (MCS), which is a birth cohort study in the UK of children born between September 2000 and January 2002, sampled at approximately 9 months old [22]. Children were initially sampled in the year 2000 and follow up data is currently available from six sweeps. Sampling was based on a cluster-stratified framework with smaller population groups being over sampled, including those in the smaller nations of the UK, as well as those in disadvantaged areas and from ethnic minority backgrounds.

These analyses use data from the fourth sweep of data collection when children were aged on average 7years old (response rate 82% and age range 6-8), the fifth sweep of data collection children were on average11 years old (response rate 81%, and age range 10-12), and the sixth sweep of data when aged on average 14years old (response rate 76%, age range 13-15). A range of topics covering the economic, social and health circumstances of children are covered alongside objective adiposity measurements. We focused on these sweeps of data as levels of active travel were low before the age of 7 years. Questionnaires and measures are administered by trained interviewers to standardised protocols and further details are available from http://www.cls.ioe.ac.uk/ [22]. Specific ethical approval was not required for this study as it involved only the secondary analyses of data that does not contain identifiable information and participants give consent for their data to be used for research purposes

Variables

This study used two objective measures of adiposity at all three time points: Body Mass Index (BMI) and percentage body fat. Height was measured using a Leicester stadiometer, and weight and body fat were measured using Tanita BF-522 W scales [22]. This provides objective data on BMI, rather than relying on participants self-reporting their height and weight.

The exposure for this study was mode of travel to school assessed by asking caregivers "*How does (child) usually travel to school?",* with prompts to give the mode with longest distance if more than one mode was used. There were seven potential responses: Public transport; School or local authority bus; minibus or coach; Car or other vehicle (including Taxi); Bicycle; Walking; and Other. We categorised these responses into three categories of private motorised (Car or other vehicle (including Taxi)), public transport (Public transport; School or local authority bus; minibus or coach) and active travel (walking and cycling). We combined walking and cycling into one overall category due to low numbers of cycling: 76 at age 7, 187 at age 11 and 147 at age 14. We excluded "Other"

For peer review only - http://bmjopen.bmj.com/site/about/guidelines.xhtml

responses (253 at age 14 (2.2%), 1,203 at age 11 (9.0%), 174 (1.3% at age 7)) due to uncertainty about what modes of travel these may be and the fact that they likely represent a heterogeneous mix of modes.

A range of covariates were included as potential confounders of the relationship between travel mode and adiposity, covering socioeconomic factors and health behaviours. Included covariates from all ages were sex; country of residence (England, Wales, Northern Ireland, Scotland); self-reported ethnicity; portions of fruit consumed per day (<3 vs. ≥3 pieces per day); breakfast consumption (seven days per week vs. not); screen use (hours per weekday watching TV or using a computer categorised as <3hours vs. ≥ 3 hours). We also created a variable to reflect days per week of sport/exercise each week (categorised as < once a week, 1–2 times a week, 3+ times a week). We also used data on whether caregivers report that their child has experienced a growth spurt, assessed using the question *"Most children have a growth spurt as they approach and during their teens. Would you say that [Cohort child's name]'s growth spurt ….?"* This question was asked only at ages 11 and 14 and we categorised this into two categories of has definitely started vs. not.

We used two markers of SEP both ascertained by self-report: household income group (equivalised based on the Organisation for Economic Co-operation and Development and categorised in five groups) and household occupational social class measured using the National Statistics Socio-Economic Classification (NS-SEC; categorised as *Not economically active; Lower; Intermediate; Managerial/Professional*).

For longitudinal analyses we included children who had complete data available from ages 7 to 14. For cross-sectional analyses at age 14 there were 11,714 children in the sample, of which we used data from 10,722 (91.5%) after excluding 253 (2.2%) for using an "other" form of transport and 739 (6.3%) for missing data on relevant covariates. For longitudinal analyses we include data from 8,432 children (82.5% of those with complete data at age 14) who had data on all relevant data from ages 7 to 14.

Analyses

The distribution of modes of travel to school across socio-demographic and behavioural characteristics was first examined descriptively.

For peer review only

Multivariate longitudinal (panel) regression models were used to assess multiple waves of data for each child. These models allow individuals to be followed over time, and also account for the clustered nature of the data (i.e. multiple responses per child over time)[23]. Linear specifications were chosen due to the continuous nature of the variables of interest. Fixed effects specifications where chosen to adjust for individual time-invariant characteristics of individuals. These for example, could be sex, genetic factors, unmeasured sociodemographic factors, early life determinants, or personality traits that could bias this relationship between transport and health. As these individual fixed effects were adjusted for, the models exploit the within-individual variation over time and measure the association between changes in travel mode and changes in adiposity whilst controlling for all (measured and unmeasured) time invariant characteristics[24][23]. We additionally controlled for time-variant confounders in the models that could bias this association over time. Confounders we controlled for were: changes in *country; highest NSSEC in household; household income; eating breakfast; frequency of eating breakfast; self-reported growth spurt; hours of TV and computer use per day; and days per week of moderate-to-vigorous physical activity.* Additionally time fixed effects were adjusted for with dummies for each wave of data to account for changes over time. The reported effect sizes from these models are interpreted as the average association between changing travel mode across all three-time points compared with changes among participants who used private transport.

First, this model was used for the two separate outcomes of BMI and percentage body fat. Second to assess differences across our two markers of SEP we used interaction tests to assess if differences were statistically different and performed stratified analyses. All analyses used cluster robust standard errors to account for hetereoskeascity and autocorrelation, and employed survey weights to correct for differential response rates between groups and over time [22][23]. We performed the hausman test of random vs. fixed effect models, which indicated ($p<0.001$) that fixed effects were superior. Analyses were conducted using Stata 14.0 and implemented using the `xtreg` package for longitudinal analysis.

Exploratory analyses

In exploratory analyses we also investigated the potential that any differentials across SEP could be caused by differing duration of journeys to school, which may both determine mode of transport and be a proxy for amounts of physical activity involved in journeys. We restricted these analyses to individuals using physically active travel modes (walking or cycling to school). We used the question *"On a typical day, how long does it take [^Cohort member's name] to get from home to school, one way?"* with options *"<5mins, 5-10 minutes, 15-30mins, 30-45mins, 45mins-1hour, 1-2 hours, 2 hours*

For peer review only - http://bmjopen.bmj.com/site/about/guidelines.xhtml

or more". We have categorised this into a binary variable of 15 minutes or more vs. less than 15 minutes each way, as a proxy for journey distance. We conducted logistic regression to assess which participants were more likely to travel 15 minutes or more to school at age 14.

Patient and Public Involvement

Patients were not involved in the design of this study

For peer review only

Results

Details of the sample and their characteristics at age 14 are given in **Table 1**. 26.1% of children used private motorised modes to travel to school, 35.3% used public transport and 38.6% used active modes. Levels of active travel ranged from 45.1% in the *Lower* NS-SEC groups to 35.2% among the M*anagerial/Professional* NS-SEC group, and were highest among those in the lowest income group (46.0%, versus 36.2% among those in the highest income households). The majority of children (5,409 64.1%) lived in England, were white (7,329, 86.9%) and had more than three hours per day of screen use (6,535, 77.5%). Modes of travel to school at previous ages are shown in **appendix tables 1 and 2.** These show for example, that 47.3% of children used private motorised modes to travel to school at age 7, compared with 43.7% at age 11. Levels of change in transport mode were high: 36.6% (3,083) of children changed mode twice between the three waves; 50.7% (4,279) changed once, and 12.7% (1,070) did not change travel mode. Overall switching between modes was more common between ages 11 and 14 (4,340 participants (51.5% of sample) changed travel mode), than between ages 7 and 11 (2,079 participants (24.7% of sample).

Results from fixed effects longitudinal regression models are given in **Table 2**. BMI increased substantially between the ages of 7 and 14 years (+4.59 kg/m^2, Confidence Interval (CI) 4.45 to 4.73 for age 14 compared with age 7). Caregiver-reported beginning of growth spurts between ages 11 and 14 was associated with increased BMI (0.22 kg/m^2, 0.14 to 0.31). Switching to eating breakfast every day was associated with lower BMI (-0.39 kg/m^2, -0.51 to -0.27), compared with not eating breakfast every day, as was moving to doing physical activity 5 days or more per week (-0.32 kg/m^2, -0.41 to -0.23). On average, after adjusting for these trends, switching from private to active travel was associated with a -0.21kg/m^2 lower BMI (95% Confidence Interval -0.31 to -0.10), while

switching from private travel to public transport was associated with a -0.13 (-0.26 to 0.01) lower BMI.

Percentage body fat was higher at age 11 (+1.06%, 0.87 to 1.25) but not at age 14 (+0.21%, -0.14 to 0.56) than at age 7. Switching to eating breakfast seven days a week was associated with a -1.34% lower body fat (-1.62 to -1.06) but the effect of switching to 3 or more hours a day of screen use did not quite reach statistical significance (+0.22%, -0.01 to 0.44). Switching to 3 to 4 days a week of physical activity was associated with a -0.25% lower body fat (CI -0.48 to -0.01) and switching to 5 or more days a week of physical activity a -1.04% lower body fat (CI -1.25 to -0.82). Switching from a private mode to an active mode of travel was associated with a -0.55% lower percentage body fat (CI -0.80 to -0.31) and switching to public transport with a -0.43% lower percentage body fat CI (-0.75 to -0.12).

Associations of changes in travel mode with BMI across our markers of SEP are shown in **Figure 1** and **Appendix Table 3.** Interaction tests did not reveal differences between income groups (p=0.130) nor NS-SEC (p=0.074). However, visual interpretation of these charts did appear to show that these relationships were most concentrated in the lower SEP groups. For example, switching to active travel was associated with a -0.32kg/m^2 lower BMI (CI -0.58 to -0.06) among those in the lowest household income group compared with -0.11kg/m^2 among the highest household income group (CI -0.24 to 0.03). Similarly switching to active travel was associated with a -0.33kg/m^2 lower BMI (CI -0.55 to -0.11) among those in the *Not Economically Active* NS-SEC group, but a -0.09kg/m^2 lower BMI (CI -0.22, 0.04) among those in *Managerial/Professional* NS-SEC group.

Results were similar for percentage body fat and are shown in **Figure 2** and **Appendix Table 4**. Interaction tests did not reveal there to be statistically significant differences in associations between income groups (p=0.908) nor NS-SEC groups (p=0.128). Switching to active travel was associated with a -0.71% lower body fat (-1.47, 0.05) among children in the lowest household income group, compared with a -0.55% lower body fat (-1.01, -0.09) among those in the highest income group. Among the *Economically Inactive* NS-SEC group, switching to an active travel mode was associated with a -0.78% lower body fat (-1.27, -0.29) compared with a -0.43% lower body fat (-0.78, -0.08) among the M*anagerial/Professional* NS-SEC group.

Exploratory analyses of the potential role of journey duration to school in findings by SEP were performed for individuals walking or cycling to school. These found that more affluent children were more likely to travel more than 15 minutes each way to school. 60.5% of children in the M*anagerial/Professional* NS-SEC group travelled 15 minutes or further compared with 51.9% of

For peer review only

those in the N*ot Economically Active* NS-SEC group (Adjusted Odds Ratio (AOR) 1.18, CI 1.04 to 1.34). 60.7% of children in the highest income households travelled 15 minutes or more to school compared with 48.6% of those in the lowest income households (AOR 1.21, 95%CI 1.03 to 1.42). Analyses interacting duration with mode of travel were restricted to only ages 11 and age 14 due to data availability, but interaction tests did not reveal a difference between the associations for walkers/cyclists more than vs. less than 15 minutes away for BMI (p= 0.259) nor body fat (p=0.452) (**Appendix Table 5**).

Discussion

This longitudinal study followed children over a key period in the lifecourse, and found that switching from private motorised modes of travel to school to active travel was associated with reduced objectively-measured adiposity. Additionally, switching to the use of public transport was associated with lower adiposity, although associations with BMI did not reach statistical significance. There was also a suggestion that these associations may be more concentrated among the most disadvantaged children, who are at greater risk of excess adiposity and relevant sequelae.

Strengths and limitations

This study used an established and representative UK birth cohort study with high quality data following up the same children over 8 years. We used two objective measures of adiposity and employed models that produce robust estimates of within-individual changes, which improves confidence in these results. We were able to adjust for unobserved time-invariant individual characteristics that can bias the relationship between travel mode and adiposity. Nonetheless, there are limitations to this research which should be considered. We rely on self-reported mode of travel to school, which while it was consistent across the study period, did not capture information on multi-mode journeys or variation in different modes across the week. This may have introduced some misclassification in our findings. Our reliance on these self-report questions is in contrast to some other research using the Millennium Cohort Study, which has used objective physical activity measures by accelerometers [25]. However, issues with using accelerometer data here include that is currently not available for the age 14 sweep; sample sizes being substantially lower for this method of data collection; and difficulties in ascertaining cycling to school. There was an average of three years between data collection points, and we do not know at what point between data collections individuals changed their travel behaviour. While we were able to consider a wide range

For peer review only

of covariates, there may be some residual confounding from time-varying factors not considered [26]. We have included some exploratory analyses of whether there are inequalities in the time taken to get to school. For these, the distance between home and school may have served as a more accurate marker of physical activity dose, although this data was not available. There is also the potential for some confounding from other lifestyle factors as, for example, individuals who switch to more active modes may be choosing to lead a healthier lifestyle in other ways also, and changes in adiposity may be occurring via alternative mechanisms than active travel.

Comparison with other research and practical implications

This study contributes to the evidence base by using a robust longitudinal examination of changes in travel modes and adiposity as well as specifically investigating whether there are differences across markers of SEP. We use a national sample and we were able to employ more robust methods, control for more potential confounding factors and follow children up for longer compared to many previous studies. The primary finding of this study is that switching to using an active mode of travel was linked to lower adiposity which add to the evidence base that active forms of travel to school are linked with more favourable levels of adiposity [11][27][28]. A review in 2011 found a limited number of studies but concluded that active travel to school was associated with greater cardiorespiratory fitness and that increased physical activity is a likely pathway [11].The second main finding of this study is that switching to public transport was linked to reduced adiposity although associations with BMI did not reach statistical significance. There has been limited consideration of the impacts of public transportation use on health, but this finding does broadly concur with a recent systematic review in adults which identified 10 relevant studies. This systematic review found that switching to public transport was associated with a -0.30 decrease in BMI, which is larger than our point estimate of -0.13, albeit in adults [29]. We also find no statistical evidence of differences between SEP groups, although there was a trend towards larger effects in more deprived groups. However, we caution against over interpretation of this finding given our restriction to only examining within-individual variation (hence reducing our statistical power) and the limited number of individuals within the study after stratifying by markers of SEP.

Nonetheless, active travel is only one potential mechanism to reduce adiposity in the population, and our findings also point to beneficial impacts of eating breakfast every day and sporting physical activity. For example, the associations identified here for switching to active forms of travel were considerably smaller than for switching to 5 or more days per week of physical activity for both BMI (-0.22kg/m^2 vs. -0.32kg/m^2) and body fat (-0.55% vs. -1.00%). Similarly, switching to being physically active ≥5 days per week was associated with larger changes in adiposity (e.g. 1.04% of body fat).

For peer review only

Both of these findings concur with a wealth of other evidence on the importance of physical activity and dietary behaviours, both using MCS data and [16][30].

While we find higher levels of active travel use among more deprived groups, the wider evidence base on physical activity by markers of deprivation presents a mixed picture. Research using objective physical activity data from the MCS found that when they were aged seven, children in more deprived areas were more likely to be obese, but were also more likely to hit recommended levels of physical activity [31]. Recent reviews have questioned whether there are differences in physical activity by deprivation, and concluded that while more affluent children do more leisure time activity, overall differences remain small for physical activity overall and for active travel specifically [32].

These findings highlight the potential for a shift to physically active modes of travel to play a role in reducing population level adiposity, and potentially to reduce health inequalities. They also reinforce calls from the World Health Organisation to develop policies which promote incorporating physical activity into daily lives [33]. However, there is mixed evidence on the how best to increase levels of active travel to school, which remains a fruitful avenue for future research [34]. Additionally, determinants of active travel are known to be wide ranging including parental concerns regarding safety, attitudes of the school and wider environmental factors [35][36][37][38][39]. Systematic reviews have found a wide range of potential determinants, including negative associations with traffic volumes, but that results were heterogeneous due to differing contexts and measures [40]. This means that there is a need for context-specific research alongside practical actions to increase the use of physically active travel modes. These will likely need to act at a number of levels, including addressing availability of safe cycle routes, school-level policies to discourage driving and national policies to improve infrastructure. Given the well-established benefits of physical activity on outcomes other than adiposity, and the potential additional benefits of increasing active travel in the form of air pollution and greenhouse gases, concerted action should be made to encourage the use of more active modes of travel to school [41].

Conclusion

For peer review only

This seven-year longitudinal study with data collected at three time points has concluded that switching to active forms of travel can have beneficial adiposity impacts at a key time in the lifecourse. These benefits may be more apparent among disadvantaged children. This research adds to the evidence base on the health impacts of active travel and suggests that targeted structural interventions may reduce inequalities in health in the UK.

Declarations

Patient and Public Involvement

Patients were not involved in the design of this study

Ethical approval

As this study involved secondary analysis the MCS ethical approval was not required. Data collection for the MCS has ethical approval from the Yorkshire and Humber ethics committee (11/YH/0203) and further details are available from http://www.cls.ioe.ac.uk/.

Consent for publication

Not applicable

Availability of data and materials

The datasets generated during and/or analysed during the current study are available in the UKDataService repository https://ukdataservice.ac.uk/

Competing interests

All authors declare that they have no competing interests

Funding

This study/project is funded by the National Institute for Health Research (NIHR) School for Public Health Research (Grant Reference Number PD-SPH-2015) as well as a NIHR Research Professorship award to Professor Millett (NIHR RP_2014-04-032). The views expressed are those of the author(s) and not necessarily those of the NIHR or the Department of Health and Social Care

Authors' contributions

AL and CM conceived of the study. AL, CM and TH devised the analyses. AL conducted the analyses with the assistance of TH. AL produced the initial draft and TH, CM, AG and YK revised this for significant intellectual content and approve the final version

Acknowledgements

We would like to acknowledge the participants of the MCS

For peer review only

Table 1: Mode of travel to school in the Millennium Cohort Study in 2014/15, age 14/15 years

		Number	Car % (number)	PT % (number)	Active % (number)
Gender	Male	4,102	24.2 (992)	35.4 (1453)	40.4 (1657)
	Female	4,330	27.9 (1206)	35.2 (1526)	36.9 (1598)
Country	England	5,409	26.9 (1455)	29.8 (1612)	43.3 (2342)
	Wales	1,182	25.5 (302)	40.3 (476)	34.2 (404)
	Scotland	958	20.0 (192)	40.5 (388)	39.5 (378)
	Northern Ireland	883	28.2 (249)	57.0 (503)	14.8 (131)
NS-SEC social class	Not economically active	2,653	27.5 (531)	37.2 (657)	35.2 (827)
	Lower	2,054	26.5 (392)	38.3 (547)	35.2 (771)
	Intermediate	1,710	22.9 (545)	32.0 (787)	45.1 (722)
	Managerial/professional	2,015	26.4 (730)	32.6 (988)	41.0 (935)
Household income group	Lowest	1,092	26.1 (285)	27.9 (305)	46 (502)
	Second lowest	1,286	23.2 (298)	36.5 (469)	40.4 (519)
	Middle	1,737	25.2 (438)	37.2 (646)	37.6 (653)
	Second highest	2,121	27.3 (578)	35.7 (757)	37.1 (786)
	Highest	2,196	27.3 (599)	36.5 (802)	36.2 (795)
Ethnic group	White	7,329	25 (1832)	36.5 (2677)	38.5 (2820)
	Mixed	64	17.2 (11)	45.3 (29)	37.5 (24)
	Indian	208	34.6 (72)	26.4 (55)	38.9 (81)
	Pakistani or Bangladeshi	468	42.7 (200)	10.9 (51)	46.4 (217)
	Black or Black British	241	18.7 (45)	49.4 (119)	32.0 (77)
	Other	122	31.1 (38)	39.3 (48)	29.5 (36)
Growth spurt	Not yet/barely started	2,952	25.4 (750)	35.9 (1060)	38.7 (1142)
	Begun or completed	5,480	26.4 (1448)	35.0 (1919)	38.6 (2113)
Breakfast consumption	< 7 days per week	3,824	25.8 (985)	35.0 (1339)	39.2 (1500)
	Every day	4,608	26.3 (1213)	35.6 (1640)	38.1 (1755)
Portions of fruit per day	≤3 portions per day	5,775	26.1 (1510)	34.5 (1994)	39.3 (2271)
	≥2 portions per day	2,655	25.9 (688)	37.1 (985)	37.0 (982)
Physical activity	≤2 days per week	2,342	27.8 (650)	34.3 (804)	37.9 (888)
	3-4 days per week	2,905	25.0 (725)	37.6 (1092)	37.5 (1088)
	≥ 5 days per week	3,185	25.8 (823)	34.0 (1083)	40.2 (1279)
TV or computer use	≤2 hours per day	1,897	26.4 (501)	38.2 (724)	35.4 (672)
	≥3 hours per day	6,535	26.0 (1697)	34.5 (2255)	39.5 (2583)
Overall		8,432	26.1 (2198)	35.3 (2979)	38.6 (3255)

PT = Public transport, NS-SEC = National Statistics Socio-Economic Classification

For peer review only

Table 2: Results from longitudinal fixed-effects regression of impacts of switching school travel mode and adiposity

		BMI		% Body fat	
		coeff	95% CI	coeff	95% CI
Mode of travel	Private transport	ref	ref	ref	ref
	Public transport	-0.13	-0.26 ;0.01	-0.43	-0.75 ;-0.12
	Walk/Cycle	-0.21	-0.31 ;-0.10	-0.55	-0.80 ;-0.31
Age	7	ref	ref	ref	ref
	11	2.44	2.37 ;2.52	1.06	0.87 ;1.25
	14	4.59	4.45 ;4.73	0.21	-0.14 ;0.56
Country	England	ref	ref	ref	ref
	Wales	0.13	-1.10 ;1.37	1.77	-0.54 ;4.08
	Scotland	0.01	-1.00 ;1.01	0.46	-1.88 ;2.79
	Northern Ireland	-0.10	-1.92 ;1.72	1.07	-3.04 ;5.19
NS-SEC social class	Not economically active	ref	ref	ref	ref
	Lower	-0.04	-0.18 ; 0.10	-0.22	-0.53 ; 0.10
	Intermediate	-0.08	-0.21 ; 0.06	-0.24	-0.58 ; 0.07
	Managerial/professional	0.08	-0.06 ; 0.22	0.16	-0.18 ; 0.50
Household income group	Lowest	ref	ref	ref	ref
	Second lowest	0.08	-0.07 ;0.23	0.41	0.05 ;0.77
	Middle	0.13	-0.03 ;0.29	0.36	-0.02 ;0.75
	Second highest	0.06	-0.11 ;0.23	0.12	-0.30 ;0.54
	Highest	-0.01	-0.20 ;0.17	0.14	-0.30 ;0.59
Growth spurt	Not yet/barely started	ref	ref	ref	ref
	Begun or completed	0.22	0.14 ;0.31	0.1	-0.11 ;0.31
Breakfast consumption	< 7 days per week	ref	ref	ref	ref
	Every day	-0.39	-0.51 ;-0.27	-1.34	-1.62 ;-1.06
Portions of fruit per day	≤3 portions per day	ref	ref	ref	ref
	≥2 portions per day	0.05	-0.04 ;0.15	0.11	-0.11 ;0.33
Physical activity	≤2 days per week	ref	ref	ref	ref
	3-4 days per week	-0.06	-0.16 ;0.04	-0.25	-0.48 ;-0.01
	≥ 5 days per week	-0.32	-0.41 ;-0.23	-1.04	-1.25 ;-0.82
TV or computer use	≤2 hours per day	ref	ref	ref	ref
	≥3 hours per day	0.07	-0.02 ;0.16	0.22	-0.01 ;0.44

Results from one separate model for BMI and one separate model for percentage body fat. Models adjusted for individual-level fixed effects and changes in: country; highest NSSEC in household; household income; eating breakfast; frequency of eating breakfast; self-reported growth spurt; hours of TV and computer use per day; and days per week of moderate-to-vigorous physical activity. Coeff = coefficient, ref = reference category

For peer review only

Figure 1: Changes in BMI and 95% Confidence Intervals by mode of travel to school

Caption

Results from longitudinal fixed-effects regression of impacts of switching school travel mode and BMI stratified by markers of Socio-Economic Position. These represent the average effect of switching to using active transport from any other mode

Figure 2: Changes in % body fat and 95% Confidence Intervals by mode of travel to school

Caption

Results from longitudinal fixed-effects regression of impacts of switching school travel mode and percentage body fat stratified by markers of Socio-Economic Position. These represent the average effect of switching to using active transport from any other mode

For peer review only

References

1 Ekelund U, Brage S, Froberg K, *et al.* TV viewing and physical activity are independently associated with metabolic risk in children: The European youth heart study. *PLoS Med* Published Online First: 2006. doi:10.1371/journal.pmed.0030488

2 Griffiths LJ, Cortina-Borja M, Sera F, *et al.* How active are our children? Findings from the millennium cohort study. *BMJ Open* Published Online First: 2013. doi:10.1136/bmjopen-2013-002893

3 Guthold R, Stevens GA, Riley LM, *et al.* Global trends in insufficient physical activity among adolescents: a pooled analysis of 298 population-based surveys with 1·6 million participants. *Lancet Child Adolesc Heal* Published Online First: 2020. doi:10.1016/S2352-4642(19)30323-2

4 Kwon S, Janz KF, Cooper A, *et al.* Tracking of accelerometry-measured physical activity during childhood: ICAD pooled analysis. *Int J Behav Nutr Phys Act* Published Online First: 2012. doi:10.1186/1479-5868-9-68

5 Hills AP, Andersen LB, Byrne NM. Physical activity and obesity in children. *Br J Sports Med* 2011;**45**:866 LP – 870. doi:10.1136/bjsports-2011-090199

6 Cooper AR, Goodman A, Page AS, *et al.* Objectively measured physical activity and sedentary time in youth: The International children's accelerometry database (ICAD). *Int J Behav Nutr Phys Act* Published Online First: 2015. doi:10.1186/s12966-015-0274-5

7 Morton KL, Corder K, Suhrcke M, *et al.* School polices, programmes and facilities, and objectively measured sedentary time, LPA and MVPA: Associations in secondary school and over the transition from primary to secondary school. *Int J Behav Nutr Phys Act* Published Online First: 2016. doi:10.1186/s12966-016-0378-6

8 Flint E, Cummins S, Sacker A. Associations between active commuting, body fat, and body mass index: population based, cross sectional study in the United Kingdom. *Br Med J* 2014;**349**. doi:10.1136/bmj.g4887

9 Roth MA, Millett CJ, Mindell JS. The contribution of active travel (walking and cycling) in children to overall physical activity levels: A national cross sectional study. *Prev Med (Baltim)* Published Online First: 2012. doi:10.1016/j.ypmed.2011.12.004

10 Colley R, Larouche R, Saunders TJ, *et al.* Associations between Active School Transport and Physical Activity, Body Composition, and Cardiovascular Fitness: A Systematic Review of 68 Studies. *J Phys Act Heal* Published Online First: 2016. doi:10.1123/jpah.2011-0345

11 Lubans DR, Boreham CA, Kelly P, *et al.* The relationship between active travel to school and health-related fitness in children and adolescents: A systematic review. Int. J. Behav. Nutr. Phys. Act. 2011. doi:10.1186/1479-5868-8-5

12 Department for Transport (2015). National Travel Survey 2014: Travel to school https://assets.publishing.service.gov.uk/government/uploads/system/uploads/attachment_data/file/476635/travel-to-school.pdf.

13 Pouliou T, Sera F, Griffiths L, *et al.* Environmental influences on children's physical activity. *J Epidemiol Community Health* Published Online First: 2015. doi:10.1136/jech-2014-204287

14 Laverty AA, Mindell JS, Webb EA, *et al.* Active travel to work and cardiovascular risk factors in the united kingdom. *Am J Prev Med* 2013;**45**:282–8. doi:10.1016/j.amepre.2013.04.012

15 Higgins, V., Dale, A., 2010. Ethnic differences in physical activity and obesity, working paper. Cathie Marsh Centre for Census and Survey Research https://lemosandcrane.co.uk/resources/University%20of%20Manchester%20-%20Ethnic%20differences%20in%20physic.

16 Goisis A, Sacker A, Kelly Y. Why are poorer children at higher risk of obesity and overweight? A UK cohort study. *Eur J Public Health* Published Online First: 2016. doi:10.1093/eurpub/ckv219

17 Cavill N, Davis A, Cope A, Corner D, 2019, Active Travel and Physical Activity Evidence Review. Sport England https://www.sportengland.org/media/13943/active-travel-full-report-evidence-review.pdf.

For peer review only

18 Su JG, Jerrett M, McConnell R, *et al.* Factors influencing whether children walk to school. *Health Place* 2013;**22**:153–61. doi:https://doi.org/10.1016/j.healthplace.2013.03.011
19 Noonan RJ, Boddy LM, Knowles ZR, *et al.* Fitness, fatness and active school commuting among liverpool schoolchildren. *Int J Environ Res Public Health* Published Online First: 2017. doi:10.3390/ijerph14090995
20 Schoeppe S, Duncan MJ, Badland H, *et al.* Associations of children's independent mobility and active travel with physical activity, sedentary behaviour and weight status: A systematic review. *J Sci Med Sport* 2013;**16**:312–9. doi:https://doi.org/10.1016/j.jsams.2012.11.001
21 Saunders LE, Green JM, Petticrew MP, *et al.* What Are the Health Benefits of Active Travel? A Systematic Review of Trials and Cohort Studies. *PLoS One* 2013;**8**:e69912. doi:10.1371/journal.pone.0069912
22 Connelly R, Platt L. Cohort profile: UK Millennium Cohort Study (mcs). *Int J Epidemiol* 2014;**43**:1719–25. doi:10.1093/ije/dyu001
23 Wooldridge JM (2010). Econometric analysis of cross section and panel data: MIT press; 2010.
24 Rabe-Hesketh S, Skrondal A (2008). Multilevel and longitudinal modeling using Stata: STATA press; 2008.
25 Griffiths LJ, Sera F, Cortina-Borja M, *et al.* Objectively measured physical activity and sedentary time: cross-sectional and prospective associations with adiposity in the Millennium Cohort Study. *BMJ Open* 2016;**6**:e010366. doi:10.1136/bmjopen-2015-010366
26 Harrison F, Goodman A, van Sluijs EMF, *et al.* Weather and children's physical activity; how and why do relationships vary between countries? *Int J Behav Nutr Phys Act* 2017;**14**:74. doi:10.1186/s12966-017-0526-7
27 Schoeppe S, Duncan MJ, Badland H, *et al.* Associations of children's independent mobility and active travel with physical activity, sedentary behaviour and weight status: A systematic review. *J Sci Med Sport* 2013;**16**:312–9. doi:https://doi.org/10.1016/j.jsams.2012.11.001
28 Faulkner GEJ, Buliung RN, Flora PK, *et al.* Active school transport, physical activity levels and body weight of children and youth: A systematic review. *Prev Med (Baltim)* 2009;**48**:3–8. doi:http://dx.doi.org/10.1016/j.ypmed.2008.10.017
29 Patterson R, Webb E, Hone T, *et al.* Associations of Public Transportation Use With Cardiometabolic Health: A Systematic Review and Meta-Analysis. *Am J Epidemiol* 2019;**188**:785–95. doi:10.1093/aje/kwz012
30 World Health Organization (2020) Report of the commission on ending childhood obesity. https://apps.who.int/iris/bitstream/handle/10665/204176/9789241510066_eng.pdf;jsessionid=F36FB7899AA415F5EDC15F7D35B4B230?sequence=1.
31 Noonan RJ, Fairclough SJ. Is there a deprivation and maternal education gradient to child obesity and moderate-to-vigorous physical activity? Findings from the Millennium Cohort Study. *Pediatr Obes* 2018;**13**:458–64. doi:10.1111/ijpo.12287
32 Stalsberg R, Pedersen AV. Are Differences in Physical Activity across Socioeconomic Groups Associated with Choice of Physical Activity Variables to Report? *Int J Environ Res Public Health* 2018;**15**:922. doi:10.3390/ijerph15050922
33 World Health Organization. Global action plan for the prevention and control of NCDs 2013-2020. Published Online First: 2013.http://www.who.int/nmh/publications/ncd-action-plan/en/
34 Larouche R, Mammen G, Rowe DA, *et al.* Effectiveness of active school transport interventions: a systematic review and update. *BMC Public Health* 2018;**18**:206. doi:10.1186/s12889-017-5005-1
35 Garnham-Lee KP, Falconer CL, Sherar LB, *et al.* Evidence of moderation effects in predicting active transport to school. *J Public Health (Oxf)* 2017;**39**:153–62. doi:10.1093/pubmed/fdw016
36 Panter J, Corder K, Griffin SJ, *et al.* Individual, socio-cultural and environmental predictors of uptake and maintenance of active commuting in children: longitudinal results from the

For peer review only

SPEEDY study. *Int J Behav Nutr Phys Act* 2013;**10**:83. doi:10.1186/1479-5868-10-83
37 Carver A, Salmon J, Campbell K, *et al.* How Do Perceptions of Local Neighborhood Relate to Adolescents' Walking and Cycling? *Am J Heal Promot* 2005;**20**:139–47. doi:10.4278/0890-1171-20.2.139
38 Panter JR, Jones AP, van Sluijs EMF, *et al.* Attitudes, social support and environmental perceptions as predictors of active commuting behaviour in school children. *J Epidemiol Community Health* 2010;**64**:41–8. doi:10.1136/jech.2009.086918
39 Morton KL, Atkin AJ, Corder K, *et al.* The school environment and adolescent physical activity and sedentary behaviour: a mixed-studies systematic review. *Obes Rev* 2016;**17**:142–58. doi:10.1111/obr.12352
40 Sharmin S, Kamruzzaman M. Association between the built environment and children's independent mobility: A meta-analytic review. *J Transp Geogr* 2017;**61**:104–17. doi:https://doi.org/10.1016/j.jtrangeo.2017.04.004
41 Woodcock J, Edwards P, Tonne C, *et al.* Public health benefits of strategies to reduce greenhouse-gas emissions: urban land transport. *Lancet* 2009;**374**:1930–43. doi:S0140-6736(09)61714-1 [pii] 10.1016/S0140-6736(09)61714-1

For peer review only

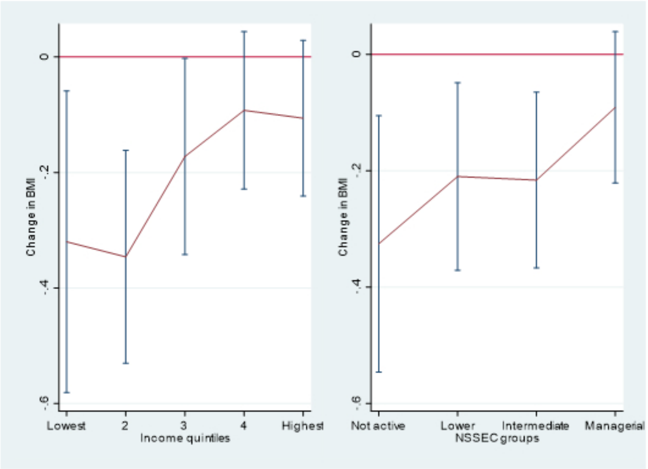

Figure 1: Changes in BMI and 95% Confidence Intervals by mode of travel to school

216x121mm (96 x 96 DPI)

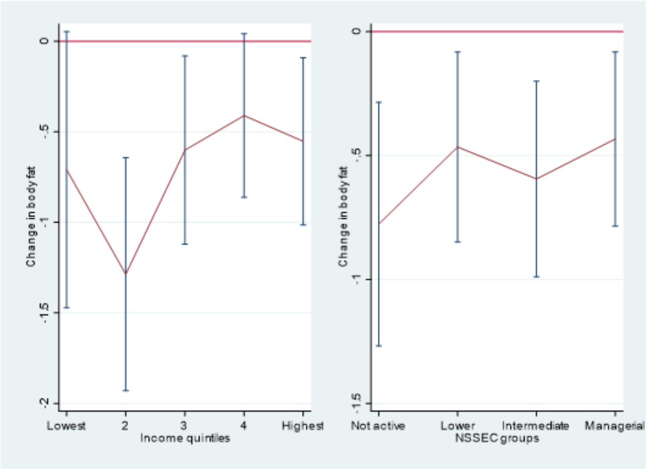

Figure 2: Changes in % body fat and 95% Confidence Intervals by mode of travel to school

216x121mm (96 x 96 DPI)

Appendix table 1: Mode of travel to school in the Millennium Cohort Study in 2011/12, age 10-11 years

		Car	PT	Active
Gender	Male	47.4	5.3	47.3
	Female	47.2	5.2	47.6
Country	England	45.5	2.6	51.9
	Wales	51.1	7.6	41.3
	Scotland	37.8	9.8	52.4
	Northern Ireland	62.9	13.9	23.1
NS-SEC social class	Not economically active	28.7	4.9	66.4
	Lower	39.7	5.7	54.6
	Intermediate	53.7	4.9	41.4
	Managerial/professional	53.7	5.1	41.2
Household income group	Lowest	34.6	5.5	59.9
	Second lowest	43.3	6.6	50.0
	Middle	47.8	5.2	47.0
	Second highest	51.1	4.5	44.4
	Highest	56.2	4.7	39.1
Ethnic group	White	48.0	5.7	46.3
	Mixed	41.6	4.7	53.7
	Indian	58.4	1.0	40.6
	Pakistani or Bangladeshi	38.1	1.1	60.8
	Black or Black British	40.5	6.2	53.3
	Other	43.5	3.3	53.3
Growth spurt*	Not yet/barely started	-	-	-
	Begun or completed	-	-	-
Breakfast consumption	< 7 days per week	42.9	7.3	49.8
	Every day	47.6	5.1	47.3
Portions of fruit per day	≤3 portions per day	46.1	5.1	48.8
	≥2 portions per day	47.6	5.3	47.1
Physical activity	≤2 days per week	48.9	4.2	46.9
	3-4 days per week	50.1	5.0	44.9
	≥ 5 days per week	46.2	5.7	48.1
TV or computer use	≤2 hours per day	47.5	5.5	47.0
	≥3 hours per day	46.4	4.2	49.4
Overall		47.3	5.3	47.4

PT = Public Transport , * N/A

For peer review only

Appendix table 2: Mode of travel to school in the Millennium Cohort Study in 2007/08, age 7-8 years

		Car	PT	Active
Gender	Male	42.6	6.6	50.9
	Female	44.7	6.9	48.4
Country	England	42.1	4.7	53.2
	Wales	47.1	8.4	44.5
	Scotland	32.2	10.1	57.8
	Northern Ireland	60.8	13.6	25.6
NS-SEC social class	Not economically active	36.8	6.8	56.4
	Lower	38.3	5.9	55.7
	Intermediate	47.8	6.4	45.8
	Managerial/professional	50.7	7.4	41.8
Household income group	Lowest	31.0	6.4	62.6
	Second lowest	40.2	8.2	51.7
	Middle	48.5	6.8	44.7
	Second highest	48.7	6.0	45.3
	Highest	46.3	6.4	47.3
Ethnic group	White	43.9	6.9	49.2
	Mixed	42.2	10.1	47.7
	Indian	55.3	2.6	42.1
	Pakistani or Bangladeshi	41.3	1.8	56.9
	Black or Black British	33.6	12.3	54.0
	Other	40.0	5.6	54.4
Growth spurt	Not yet/barely started	45.0	6.5	48.5
	Begun or completed	42.3	7.0	50.7
Breakfast consumption	< 7 days per week	40.0	8.5	51.5
	Every day	44.2	6.5	49.3
Portions of fruit per day	≤3 portions per day	42.3	7.3	50.5
	≥2 portions per day	44.3	6.5	49.2
Physical activity	≤2 days per week	44.7	6.8	48.5
	3-4 days per week	44.4	6.6	49.0
	≥ 5 days per week	42.7	6.7	50.6
TV or computer use	≤2 hours per day	44.5	7.1	48.4
	≥3 hours per day	39.9	5.0	55.1
Overall		43.7	6.7	49.6

For peer review only

Appendix Table 3: Results from longitudinal fixed-effects regression of impacts of switching school travel mode and BMI stratified by markers of Socio-Economic Position

NSSEC groups	Coefficient	Lower CI	Upper CI
Managerial/professional	-0.09	-0.22	0.04
Self-employed intermediate	-0.22	-0.37	-0.07
Lower semi-routine routine	-0.21	-0.37	-0.05
Not economically active	-0.33	-0.55	-0.11
Household income groups			
Highest quantile income	-0.11	-0.24	0.03
Fourth quantile	-0.09	-0.23	0.04
Third quantile	-0.17	-0.34	0.00
Second quantile	-0.35	-0.53	-0.16
Lower quantile income	-0.32	-0.58	-0.06

NSSEC = National Statistics Socio-economic Classification, CI = Confidence Interval

Models adjusted for individual-level fixed effects and changes in: country; highest NSSEC in household; household income; eating breakfast; frequency of eating breakfast; self-reported growth spurt; hours of TV and computer use per day; and days per week of moderate-to-vigorous physical activity.

Appendix Table 4: Results from longitudinal fixed-effects regression of impacts of switching school travel mode and percentage body fat stratified by markers of Socio-Economic Position

NSSEC groups	Coefficient	Lower CI	Upper CI
Managerial/professional	-0.43	-0.78	-0.08
Self-employed intermediate	-0.59	-0.99	-0.20
Lower semi-routine routine	-0.47	-0.85	-0.08
Not economically active	-0.78	-1.27	-0.29
Household income groups			
Highest quantile income	-0.55	-1.01	-0.09
Fourth quantile	-0.41	-0.86	0.04
Third quantile	-0.60	-1.12	-0.08
Second quantile	-1.29	-1.93	-0.64
Lower quantile income	-0.71	-1.47	0.05

Models adjusted for individual-level fixed effects and changes in: country; highest NSSEC in household; household income; eating breakfast; frequency of eating breakfast; self-reported growth spurt; hours of TV and computer use per day; and days per week of moderate-to-vigorous physical activity.

For peer review only

Appendix table 5: Fully adjusted odds ratios travelling 15 minutes or more each way by walking or cycling

		%	OR	Lower CI	Upper CI
Gender	Male	42.2	ref	ref	ref
	Female	45.1	1.14	1.00	1.30
Country	England	45.2	ref	ref	ref
	Wales	38.5	0.65	0.53	0.79
	Scotland	41.2	0.74	0.61	0.91
	Northern Ireland	36.6	0.60	0.43	0.83
NS-SEC social class	Not economically active	39.7	ref	ref	ref
	Lower	43.8	1.10	0.91	1.33
	Intermediate	44.5	1.07	0.87	1.32
	Managerial/professional	47.0	1.20	0.98	1.47
Household income group	Lowest	38.2	ref	ref	ref
	Second lowest	43.8	1.00	0.80	1.25
	Middle	44.9	0.98	0.77	1.23
	Second highest	44.9	0.88	0.69	1.11
	Highest	46.0	0.86	0.66	1.11
Ethnic group	White	46.2	ref	ref	ref
	Mixed	53.8	1.30	0.67	2.53
	Indian	36.4	0.60	0.41	0.88
	Pakistani or Bangladeshi	23.9	0.30	0.23	0.40
	Black or Black British	38.9	0.66	0.45	0.97
	Other	32.3	0.53	0.31	0.90
Growth spurt	Not yet/barely started	43.3	ref	ref	ref
	Begun or completed	44.2	0.99	0.87	1.13
Breakfast consumption	< 7 days per week	43.3	ref	ref	ref
	Every day	44.0	1.07	0.94	1.22
Portions of fruit per day	≤3 portions per day	43.4	ref	ref	ref
	≥2 portions per day	44.4	1.00	0.87	1.16
Physical activity	≤2 days per week	42.7	ref	ref	ref
	3-4 days per week	45.6	1.14	0.97	1.34
	≥ 5 days per week	42.5	0.98	0.83	1.15
TV or computer use	≤2 hours per day	42.5	ref	ref	ref
	≥3 hours per day	43.9	1.01	0.86	1.18

For peer review only

STROBE 2007 (v4) Statement—Checklist of items that should be included in reports of *cohort studies*

Section/Topic	Item #	Recommendation	Reported on page #
**Title and abstract**	1	(*a*) Indicate the study's design with a commonly used term in the title or the abstract	1
		(*b*) Provide in the abstract an informative and balanced summary of what was done and what was found	1 - 2
**Introduction**			
Background/rationale	2	Explain the scientific background and rationale for the investigation being reported	4 - 5
Objectives	3	State specific objectives, including any prespecified hypotheses	5
**Methods**			
Study design	4	Present key elements of study design early in the paper	4 - 5
Setting	5	Describe the setting, locations, and relevant dates, including periods of recruitment, exposure, follow-up, and data collection	5 - 6
Participants	6	(*a*) Give the eligibility criteria, and the sources and methods of selection of participants. Describe methods of follow-up	5 - 6
		(*b*) For matched studies, give matching criteria and number of exposed and unexposed	n / a
Variables	7	Clearly define all outcomes, exposures, predictors, potential confounders, and effect modifiers. Give diagnostic criteria, if applicable	5 - 7
Data sources/ measurement	8*	For each variable of interest, give sources of data and details of methods of assessment (measurement). Describe comparability of assessment methods if there is more than one group	*6*
Bias	9	Describe any efforts to address potential sources of bias	6
Study size	10	Explain how the study size was arrived at	5
Quantitative variables	11	Explain how quantitative variables were handled in the analyses. If applicable, describe which groupings were chosen and why	5 - 6
Statistical methods	12	(*a*) Describe all statistical methods, including those used to control for confounding	5 – 6
		(*b*) Describe any methods used to examine subgroups and interactions	6
		(*c*) Explain how missing data were addressed	6
		(*d*) If applicable, explain how loss to follow-up was addressed	6
		(*e*) Describe any sensitivity analyses	6
**Results**			

For peer review only

Participants	13*	(a) Report numbers of individuals at each stage of study—eg numbers potentially eligible, examined for eligibility, confirmed eligible, included in the study, completing follow-up, and analysed	6
		(b) Give reasons for non-participation at each stage	5 – 6
		(c) Consider use of a flow diagram	n / a
Descriptive data	14*	(a) Give characteristics of study participants (eg demographic, clinical, social) and information on exposures and potential confounders	Table 1
		(b) Indicate number of participants with missing data for each variable of interest	Table 1
		(c) Summarise follow-up time (eg, average and total amount)	5 - 6
Outcome data	15*	Report numbers of outcome events or summary measures over time	7
Main results	16	(*a*) Give unadjusted estimates and, if applicable, confounder-adjusted estimates and their precision (eg, 95% confidence interval). Make clear which confounders were adjusted for and why they were included	7
		(*b*) Report category boundaries when continuous variables were categorized	7 – 9
		(*c*) If relevant, consider translating estimates of relative risk into absolute risk for a meaningful time period	8
Other analyses	17	Report other analyses done—eg analyses of subgroups and interactions, and sensitivity analyses	8
**Discussion**			
Key results	18	Summarise key results with reference to study objectives	9
**Limitations**			
Interpretation	20	Give a cautious overall interpretation of results considering objectives, limitations, multiplicity of analyses, results from similar studies, and other relevant evidence	9
Generalisability	21	Discuss the generalisability (external validity) of the study results	10
**Other information**			
Funding	22	Give the source of funding and the role of the funders for the present study and, if applicable, for the original study on which the present article is based	12

*Give information separately for cases and controls in case-control studies and, if applicable, for exposed and unexposed groups in cohort and cross-sectional studies.

Note: An Explanation and Elaboration article discusses each checklist item and gives methodological background and published examples of transparent reporting. The STROBE checklist is best used in conjunction with this article (freely available on the Web sites of PLoS Medicine at http://www.plosmedicine.org/, Annals of Internal Medicine at http://www.annals.org/, and Epidemiology at http://www.epidem.com/). Information on the STROBE Initiative is available at www.strobe-statement.org.

For peer review only
